# Extracellular Matrix/Glycopeptide Hybrid Hydrogel as an Immunomodulatory Niche for Endogenous Cardiac Repair after Myocardial Infarction

**DOI:** 10.1002/advs.202301244

**Published:** 2023-06-15

**Authors:** Pengxu Kong, Jing Dong, Wenchao Li, Zefu Li, Rui Gao, Xiang Liu, Jingrong Wang, Qi Su, Bin Wen, Wenbin Ouyang, Shouzheng Wang, Fengwen Zhang, Shuyi Feng, Donglin Zhuang, Yongquan Xie, Guangzhi Zhao, Hang Yi, Zujian Feng, Weiwei Wang, Xiangbin Pan

**Affiliations:** ^1^ Department of Structural Heart Disease National Center for Cardiovascular Disease China and State Key Laboratory of Cardiovascular Disease Fuwai Hospital Chinese Academy of Medical Sciences and Peking Union Medical College National Health Commission Key Laboratory of Cardiovascular Regeneration Medicine National Clinical Research Center for Cardiovascular Diseases Beijing 100037 China; ^2^ Department of Pediatric Cardiac Surgery Huazhong Fuwai Hospital Zhengzhou University People's Hospital Henan Provincial People's Hospital Zhengzhou 450000 China; ^3^ Tianjin Key Laboratory of Biomaterial Research Institute of Biomedical Engineering Chinese Academy of Medical Sciences and Peking Union Medical College Tianjin 300192 China; ^4^ Department of Polymer Science and Engineering Key Laboratory of Systems Bioengineering (Ministry of Education) School of Chemical Engineering and Technology Tianjin University Tianjin 300072 China; ^5^ Department of Cardiac Surgery Beijing Chao‐Yang Hospital Capital Medical University Beijing 100020 China; ^6^ Key Laboratory of Innovative Cardiovascular Devices Chinese Academy of Medical Sciences Beijing 100037 China; ^7^ Department of Thoracic Surgery National Cancer Center/National Clinical Research Center for Cancer/Cancer Hospital Chinese Academy of Medical Sciences and Peking Union Medical College Beijing 100021 China

**Keywords:** extracellular matrix, glycopeptide, hydrogel, macrophages polarization, tissue regeneration

## Abstract

The treatment of myocardial infarction (MI) remains a substantial challenge due to excessive inflammation, massive cell death, and restricted regenerative potential, leading to maladaptive healing process and eventually heart failure. Current strategies of regulating inflammation or improving cardiac tissue regeneration have limited success. Herein, a hybrid hydrogel coassembled by acellular cardiac extracellular matrix (ECM) and immunomodulatory glycopeptide is developed for endogenous tissue regeneration after MI. The hydrogel constructs a niche recapitulating the architecture of native ECM for attracting host cell homing, controlling macrophage differentiation via glycopeptide unit, and promoting endotheliocyte proliferation by enhancing the macrophage‐endotheliocyte crosstalk, which coordinate the innate healing mechanism for cardiac tissue regeneration. In a rodent MI model, the hybrid hydrogel successfully orchestrates a proreparative response indicated by enhanced M2 macrophage polarization, increased angiogenesis, and improved cardiomyocyte survival, which alleviates infarct size, improves wall thicknesses, and enhances cardiac contractility. Furthermore, the safety and effectiveness of the hydrogel are demonstrated in a porcine MI model, wherein proteomics verifies the regulation of immune response, proangiogenesis, and accelerated healing process. Collectively, the injectable composite hydrogel serving as an immunomodulatory niche for promoting cell homing and proliferation, inflammation modulation, tissue remodeling, and function restoration provides an effective strategy for endogenous cardiac repair.

## Graphical abstract

Here, this work develops extracellular matrix/glycopeptide hybrid hydrogel as an immunomodulatory niche for endogenous cardiac repair after myocardial infarction through coordinating the innate healing mechanisms, including homing of sufficient host cells, elaboration and control of cell differentiation, and promotion of cell proliferation and neo‐tissue formation, which can be summarized as “HELP” (Homing, Elaboration, and Proliferation) regenerative strategy.

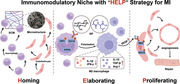

## 1 Introduction

Myocardial infarction (MI) is one of the leading causes of mortality worldwide, resulting in huge social and economic burden.^[^ ^1^ ^]^ Despite advances in surgical therapies of revascularization such as percutaneous coronary intervention and coronary artery bypass grafting, cardiomyocyte death and extracellular matrix (ECM) degradation induced by aggravated inflammation, ischemia and hypoxia remains irreversible, leading to maladaptive healing process and eventually heart failure. Unfortunately, no current treatment strategy provides desired clinical effects for post‐MI cardiac tissue regeneration.

The cardiac repair after MI involves three overlapping phases, including inflammatory, proliferative, and healing phases,^[^ ^2^ ^]^ where inflammation plays a central role.^[^ ^3^ ^]^ A properly orchestrated inflammatory process facilitates necrotic tissue removal, maintains homeostasis, promotes neo‐angiogenesis, and expedites myocardium healing, while a dysregulated inflammatory response exacerbates cardiomyocyte apoptosis, aggravates fibrosis, induces adverse remodeling and finally leads to heart failure.^[^ ^4^ ^]^ Over the past decades, therapeutic pharmacological interventions ranging from broad immunosuppression to immunomodulatory approaches targeting specific cell types or factors attempted to prevent excessive post‐MI inflammation.^[^ ^5^ ^]^ Despite encouraging results were shown in animal models, clinical trials have yielded conflicting outcomes.^[^ ^6^ ^]^ Current immunoregulatory strategies separately focus on a single offsetting factor with limited modulating capacity for the inflammatory cascade after MI, which could not fully activate the following reparative phase for cardiac healing. Moreover, long‐term systemic administration of high‐dose immunomodulatory drugs could cause severe complications owing to off‐target effects, such as fetal infection, tumor formation and gastrointestinal events.^[^ ^5^, ^7^ ^]^ Thus, none of these drugs have been recommended in clinical guidelines so far.

Alternatively, engineered biomaterials without the addition of cytokines, drugs or cells provide a promising approach for endogenous cardiac tissue regeneration post MI through “stimulating” the innate healing mechanism to control cell functions for cardiac healing.^[^ ^8^ ^]^ Biomaterials designed with precise control over biophysical and biochemical cues could be implanted in situ for host cell homing, modulation and tissue regeneration.^[^ ^8^, ^9^ ^]^ Representatively, injectable hydrogels, which possess 3D microstructure and abundant water, have been utilized for endogenous cardiac repair.^[^ ^10^ ^]^ An injectable alginate hydrogel has been tested in clinical trials to stabilize the degradation of ECM and alleviate ventricular wall stress, but fails to improve the reconstruction of cardiac structure after MI in the long‐term outcome.^[^ ^11^ ^]^ Another representative attempt is a hydrogel derived from decellularized porcine myocardium that can be applied in situ by minimally invasive injection and act as native ECM substitution to provide 3D fibrillar network and binding ligands for cell adhesion and infiltration.^[^ ^12^ ^]^ The first‐in‐man clinical trial demonstrated the feasibility and safety of the hydrogel in post‐MI patients, which, however, did not observe significant improvement of ventricular remodeling after 6‐month follow‐up,^[^ ^13^ ^]^ probably because the limited control over the initiation of proreparative immune response. Therefore, rational design of biomaterials that can trigger an orchestrated pro‐regenerative response on its own to boost the endogenous cardiac repair after MI remains to be elucidated.

**Figure 1:**
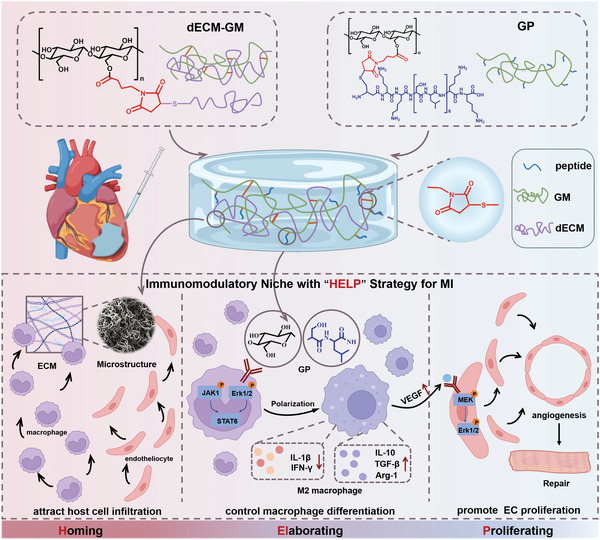
Schematic illustration of synthesis of dECM/GP hydrogel and application for post‐MI cardiac repair. To prepare dECM/GP hydrogel, MAL‐GM is synthesized through esterification. Then KK peptide and dECM are chemically conjugated to MAL‐GM by Michael addition between sulfhydryl and maleimide group. dECM/GP hydrogel conforming to “HELP” strategy can act as an immunomodulatory niche by attracting host cell infiltration with microstructure and ECM ingredients, controlling macrophage differentiation with GP unit, and promoting endotheliocyte (EC) proliferation through VEGF‐MAPK pathway, as a whole for cardiac repair post MI.

Here, we fabricated an injectable hybrid hydrogel as an immunomodulatory niche for endogenous cardiac repair after MI through coordinating the innate healing mechanisms, including homing of sufficient host cells, elaboration and control of cell differentiation, and promotion of cell proliferation and neo‐tissue formation, which could be summarized as “HELP” (Homing, Elaboration, and Proliferation) regenerative strategy. As is shown in **Figure** **1**, the injectable hybrid dECM/GP with porous fibrillar structure is fabricated based on decellularized ECM (dECM) from porcine heart and synthesized glycopeptide to restore native structure similar with autogenous heart tissue and offer binding ligands for host cell homing, adhesion, and infiltration. Glucomannan (GM) is conjugated with proangiogenic peptide (CKKSLSLSLSLSLSLKK, namely KK peptide) by thiol‐ene Michael addition to obtain the glycopeptide (donated as GP) to switch the immune response from inflammation to a preparative paradigm by polarization of recruited macrophages into M2‐type. Then, the hybrid dECM/GP hydrogel as an immunomodulatory niche can significantly promote endotheliocyte proliferation by enhancing macrophage‐endotheliocyte crosstalk. Our results demonstrate that in rat MI models, without additional stem cells, cytokines or drugs, dECM/GP hydrogel significantly improves the endogenous cardiac repair by regulating M2 macrophage polarization, promoting the vessel formation, and rescuing cardiomyocytes. Furthermore, the safety and effectiveness of the hydrogel are demonstrated in a porcine MI model, wherein proteomics verifies that the positive regulation of endotheliocyte migration, regulation of immune response, proangiogenesis and accelerated wound healing are activated in the immunomodulatory niche. Altogether, immunomodulatory dECM/GP hydrogel conforming to “HELP” strategy shows safety and effectiveness in promoting post‐MI cardiac tissue regeneration, holding great promise for MI treatment and clinical translation.

## 2 Results and Discussion

### 2.1 Preparation and Characterization of dECM/GP Hydrogel

The cardiac ECM is a complex architectural network consisting of structural and nonstructural proteins, which not only provides mechanical support, but also transduces key signals and orchestrates cellular responses.^[^ ^14^ ^]^ Meanwhile, interactions between ECM and immune cells regulate key processes in post‐MI cardiac tissue repair, including inflammation, fibrosis, and angiogenesis.^[^ ^15^ ^]^ Herein, in this study, to restore ECM constitutes and eradicate immunogenicity, porcine cardiac tissue was isolated from left ventricle and was dissected into small fragments, followed by decellularization, lyophilization, and mill into a powder (Figure S1A, Supporting Information).^[^ ^16^ ^]^ Decellularization was confirmed by Masson trichrome staining (Figure S1B, Supporting Information). Next the powder was digested in an acid environment and neutralized with NaOH to obtain the dECM hydrogel. The mechanical properties of dECM hydrogel were investigated by rheology analysis. Figure S1C (Supporting Information) shows that the storage modulus (*G*′) of dECM hydrogel increased from mean value of 84–771 Pa with the concentration of ECM increasing from 10 to 60 mg mL^−1^. Considering the mechanical strength and manufacture, the concentration of 50 mg mL^−1^ was determined for the next proof‐of‐concept studies.

To further improve the mechanical stability and bioactivity of elaborating cell differentiation and promoting proliferation for post‐MI tissue regeneration, glycopeptide with biofunctions of immunoregulation and proangiogenesis was further introduced. GM is a kind of polysaccharides composed of repeating mannose and glucose units, exhibiting highly affinitive to specific cell receptors like mannose receptors in macrophages,^[^ ^17^ ^]^ which has been widely used as scaffolds for tissue regeneration.^[^ ^18^ ^]^ Additionally, KK is a self‐assembling amphiphilic peptide,^[^ ^19^ ^]^ which can elicit angiogenesis in vivo.^[^ ^20^ ^]^ Inspired by the structure of glycan components in ECM, the glycopeptide was prepared through pendant grafting KK peptide on the backbone of GM. Firstly, maleimide functionalized GM (MAL‐GM) was synthesized through esterification. The ^1^H‐nuclear magnetic resonance (^1^H‐NMR) spectra of MAL‐GM showed an extra peak at 1.2 parts per million (ppm) (Figure S2, Supporting Information), which confirmed the successful synthesis. The ultraviolet visible (UV–vis) spectrum showed that the maximum absorbance peak of MAL‐GM and 4‐maleimidobutyric acid occurred at a wavelength of 300 nm (Figure S3, Supporting Information), consistent with previous study,^[^ ^21^ ^]^ while the GM did not result an absorbance peak above 290 nm. The degree of substitution of maleimide group in MAL‐GM was 5.08% calculated by UV–vis spectrum.

**Figure 2:**
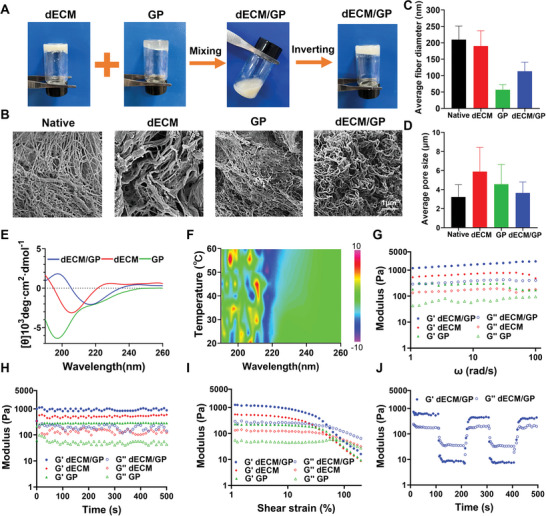
Fabrication and characterization of dECM/GP hydrogel. A) Process of dECM/GP hydrogel fabrication. B) Representative SEM images of native cardiac decellularized tissue, dECM, GP and dECM/GP hydrogels. Fiber diameter C) and pore size D) in native tissue, dECM, GP and dECM/GP hydrogels. E) CD spectrum of dECM, GP and dECM/GP hydrogels. F) CD spectrum of dECM/GP hydrogel as a function of temperature. Rheology of dECM, GP and dECM/GP hydrogels as a function of angular frequency G), time H), and shear strain I). J) Self‐healing property of dECM/GP hydrogel.

To prepare dECM/GP hydrogel, KK peptide was chemically conjugated to MAL‐GM by Michael addition between sulfhydryl and maleimide group. The KK peptide was expected to self‐assemble into nanofibrous structure and crosslink the GM. Moreover, the cystine residue of dECM also could react with maleimide to reinforce the hydrogel network. After mixing ECM (50 mg mL^−1^), MAL‐GM (40 mg mL^−1^) and peptide (20 mg mL^−1^) solutions at the volume ratio of 1:1:1, the dECM/GP hydrogel was formed upon vortex (**Figure** **2**A). The concentrations were according to rheology of dECM (Figure S1C, Supporting Information) and previous studies.^[^ ^20^, ^22^ ^]^ The sol‐to‐gel transition of hydrogels was examined by tube inverting test. As is shown in Figure 2A, after mixing, the dECM/GP solutions turned into solid gels after incubation for 50 min at room temperature, attributed to Michael addition between sulfhydryl group (from dECM and peptide) and maleimide group (from MAL‐GM), intramolecular interaction and self‐assembling of the amphiphilic peptides.

Microstructure is one of the determinants involving in bioactivities and functions of biomaterials, which affects host biological responses including host cell homing and differentiation.^[^ ^23^ ^]^ Representative images of scanning electron microscope (SEM) showed that the native porcine cardiac decellularized tissue was a highly porous and compact microarchitecture, with a mean pore size of 3.2 µm and a mean fiber diameter of 209.5 nm (Figure 2B–D). The fiber diameter was distributed diversely ranging from tens to hundreds nm, owing to complex ingredients of proteins and polysaccharides in natural tissue. Extracted dECM hydrogel had a comparable fiber size (190.2 nm), while the microstructure was loosened with the pore size increased to 5.9 µm, owing to the digestion. The morphology of GP fiber (56.7 nm) was rather uniform (ranging from 40 to 90 nm) and significantly smaller than that of native cardiac tissue, due to the self‐assembled nanofibrous structure of KK peptide.^[^ ^20^ ^]^ After conjugating with ECM, the hybrid hydrogel displayed a more compact network with a fiber diameter (113.2 nm) and a smaller pore size (3.6 µm). Collectively, dECM/GP hydrogel consisted of a highly porous and compact microstructure mimicking the native cardiac matrix.

Subsequently, circular dichroism (CD) spectroscopy was utilized to further investigate the secondary conformation of dECM/GP hydrogel. As is shown in Figure 2E, dECM hydrogel exhibited a negative peak at 206 nm and a positive curve at <195 nm, which was possibly attributed to a composite structure of *α*‐helix, *β*‐sheet, and random roil of various proteins in dECM. The GP hydrogel displayed a simple random roil signature with a negative peak at 196 nm driven by the amphiphilic, self‐assembly peptide.^[^ ^19^ ^]^ dECM/GP hydrogel displayed a CD spectrum characteristic of a typical antiparallel *β*‐sheet with a positive band at 197 nm and a negative band at 217 nm, which is a typical protein structure abundant in native ECM. The secondary structure properties were further evaluated by Bestsel analysis.^[^ ^24^ ^]^ Figure S4 (Supporting Information) shows that dECM/GP hydrogel was predicted to possess greater proportion of antiparallel *β*‐sheet structure (48.3%) compared with dECM (43.3%) and GP (31.9%) hydrogel. Moreover, as the temperature increased, no significant change was observed in the ellipticity of CD spectra and the overall profile was maintained over 20–60 °C (Figure 2F), which indicated a great temperature stability of the as‐formed secondary structure of dECM/GP hydrogel.

### 2.2 Rheological Characterization of dECM/GP

After MI, the myocardium is vulnerable to overloaded stress due to impaired ECM, which aggravates the process of heart failure. Therefore, the hydrogel is expected to provide proper ECM mechanical stability against hemodynamic pressure for constructing a persistent cell regulation niche. Oscillatory shear rheology under various mechanical measurement factors was utilized to evaluate the mechanical properties of dECM/GP hydrogel. As shown in Figure 2G, the values of *G*′ were constantly greater than loss modulus (*G*″) within the angular frequency sweep of 1–100 rad s^−1^ for dECM, GP, and dECM/GP hydrogels, indicating the formation of elastic and solid hydrogels. The *G*′ of dECM/GP was significantly greater than its single component (mean value of 1659 Pa of dECM/GP vs 269 Pa of GP vs 679 Pa of dECM) due to the formation of chemical crosslink network. Previous studies have demonstrated that soft substrates (≈Pa–kPa) favor cell infiltration and proliferation.^[^ ^25^ ^]^ Figure 2H shows that the value of *G*′ of dECM/GP hydrogel ranged between 800 and 1200 Pa and remained stable throughout the time swap, indicating that the strength of dECM/GP hydrogel could not only provide steady mechanical support but also would not perturb cell bioactivity. The strain sweep was utilized to investigate the linear viscoelastic region and the gel–sol transition point. Figure 2I shows that all of the three hydrogels exhibited non‐Newtonian shear thinning behavior, with the values of *G*′ decreased greater than *G*″ when the shear strain increased. The gel‐to‐sol phase transition of dECM/GP hydrogels occurred at the shear strain of 52%, indicating that the gels could be easily injected through syringe and catheter. The self‐healing ability of the hydrogel was evaluated by step‐strain measurement. Figure 2J shows that dECM/GP was a solution statue when applying a large strain of 55%. However, when the strain decreased to 2%, dECM/GP hydrogel could quickly recover to a solid status. The rapid self‐healing ability rendered fast gelation after injection into heart. These results confirmed that dECM/GP hydrogel with mechanical stability, shear thinning behavior and self‐healing capability was suitable for cardiac injection. dECM/GP hydrogel with a stiffness of ≈1000 Pa not only rendered it supporting strength for the ECM stability, but also could favor cellular infiltration, angiogenesis and expression of vascular endothelial growth factor receptor 2 (VEGFR2).^[^ ^26^ ^]^

### 2.3 Cytocompatibility of dECM/GP Hydrogel

The biocompatibility and bioactivity of hydrogels were first evaluated in cellular level. The cytocompatibility was tested by coculturing L929 cells (a mouse fibroblast cell line) and H9C2 cells (a rat myocardial cell line) with dECM, GP and dECM/GP hydrogels. After 3 days, cells cocultured with dECM/GP hydrogel showed high viability determined by CCK‐8 assay (Figure S5A,C, Supporting Information). The cytotoxicity was further visually investigated by live/dead staining assay. As is shown in Figure S5B,D (Supporting Information), after culturing for 1 and 3 days, a large number of survival L929 and H9C2 cells (green fluorescence) and almost no dead cells (red fluorescence) were detected, which were comparable with control group. Also, the amount of survival cells continually increased during 3 days.

Blood compatibility is important for cardiovascular implants. Hemolysis test showed that the hemolysis rate was low (0.16%) after treatment of the dECM/GP (Figure S6A, Supporting Information). Blood coagulation assay showed that dECM/GP did not significantly trigger thrombogenesis (Figure S6B, Supporting Information). The blood colt index (BCI) of dECM/GP was also tested, which quantitatively evaluates the ability to induce thrombosis and is inversely proportional to the thrombogenic effect.^[^ ^27^ ^]^ The results demonstrated that dECM/GP displayed long clotting time and was similar with the blank control (Figure S6C, Supporting Information). These results confirmed that dECM/GP hydrogel was blood compatible and would not induce hemolysis and thrombosis when injected into the heart.

### 2.4 M2 Macrophage Polarization by dECM/GP Hydrogel

**Figure 3:**
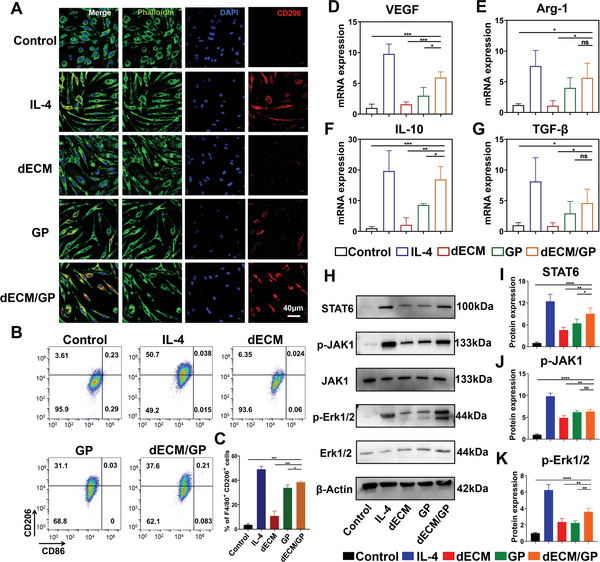
dECM/GP facilitated M2‐type macrophage polarization. A) Representative images of BMDMs treated with IL‐4, dECM, GP, and dECM/GP (green: F‐actin; blue: nuclear; red: CD206). B,C) Quantification of CD206 and CD86 expression in F4/80^+^ BMDMs (*n* = 3). Relative mRNA expression of VEGF D), Arg‐1 E), IL‐10 F), and TGF‐*β* G) in treated BMDMs (*n* = 3). H–K) Relative protein expression of STAT6 I), p‐JAK J) and p‐ERK1/2 K) in BMDMs determined by western blotting (*n* = 4).

Macrophages are closely involved in post‐MI inflammation and cardiac repair.^[^ ^28^ ^]^ Macrophages can be polarized into two classically described group: proinflammatory M1 (CD86^+^) or proreparative M2 (CD206^+^) phenotype, which may be influenced by physical and biochemical properties of biomaterials.^[^ ^29^ ^]^ Previous studies suggest that GM and KK peptide display unique bioactivity in immunoregulation and vascularization.^[^ ^20^, ^22^ ^]^ GM acting as a mannose receptor (CD206) ligand with high affinity can trigger intracellular signal transduction and prime M2 phenotype macrophages polarization. And KK peptide can provoke immune cell recruitment and induce release of chemokines for angiogenesis in vivo. Moreover, the 3D network structure of dECM/GP hydrogel may provide a favorable microenvironment for cell proliferation and differentiation. Therefore, we hypothesized that dECM/GP hydrogel could act as an immunomodulatory niche by promoting polarization of M2 phenotype macrophage. Bone marrow‐derived macrophages (BMDMs) from C57BL/6 mice were used to investigate the polarization effect of dECM/GP hydrogel. Interleukin‐4 (IL‐4), a well‐established stimulus for M2 macrophages, was used as a positive control. First, the morphology was determined by immunofluorescence staining, with FITC‐conjugated phalloidine to label the F‐actin and APC‐CD206 to identify M2 type macrophages. As is shown in **Figure** **3**A, dECM/GP group displayed stronger intensity of CD206 compared with GP and dECM groups, indicating that dECM/GP might stimulate M2 macrophage polarization. Next, flow cytometry was utilized to quantitatively verify the polarization effect (Figure S7, Supporting Information). Figure 3B,C shows that dECM/GP hydrogel induced significantly greater proportion of M2 type macrophage (mean percentage of 38.5%) compared with dECM (10.85%) and GP (33.8%) hydrogels (*p* < 0.05). All the three hydrogels induced negligible M1 type polarization (<0.5%), indicating that the hydrogels would not result in proinflammatory response. Reverse transcription‐polymerase chain reaction (RT‐PCR) was employed to evaluate the expression of M2‐related genes. Figure 3D–G shows that the level of M2 macrophages related mRNA expression (including Arg‐1, VEGF, IL‐10, and TGF‐*β*) in dECM/GP and GP group was upregulated compared with control and dECM groups, which confirmed the M2 macrophages polarization effect of dECM/GP hydrogels.

We further investigated the signal transduction pathway for macrophage polarization through western blot. Transcription factor signal transducer and activator of transcription 6 (STAT6), a key signaling protein of M2 macrophage activation, was significantly upregulated in GP and dECM/GP groups (Figure 3H,I). Previous studies show that M2 macrophage polarization can be activated through Janus kinase type 1 (JAK1) phosphorylation.^[^ ^30^ ^]^ As shown in Figure 3H,J, the phosphorylated JAK1 was significantly upregulated by IL‐4, GP, and dECM/GP stimulus. However, no difference was detected between dECM/GP and GP groups. Considering the higher percentage of M2 macrophage in dECM/GP treatment, we hypothesized that the macrophages in dECM/GP treatment group might activate through additional pathways. Extracellular signal regulated kinase (Erk) 1/2, a crucial regulator of cell differentiation,^[^ ^31^ ^]^ was further studied. Figure 3H shows that the expression of phosphorylated Erk1/2 was significantly upregulated by dECM, GP and dECM/GP treatment compared with control. However, the relative value of p‐Erk1/2 in dECM/GP treatment was 1.8‐fold to that in dECM and GP groups (*p* < 0.05), probably because the greater matrix stiffness and microstructure modulated cell differentiation pathway. These results suggested that dECM/GP hydrogel could effectively prime macrophages toward M2 phenotype by activation of JAK1 and Erk1/2 pathway, which might promote cardiac tissue regeneration by proreparative immunomodulation.

### 2.5 Promotion of Migration and Tube Formation of Endothelial Cells by dECM/GP Hydrogel

**Figure 4:**
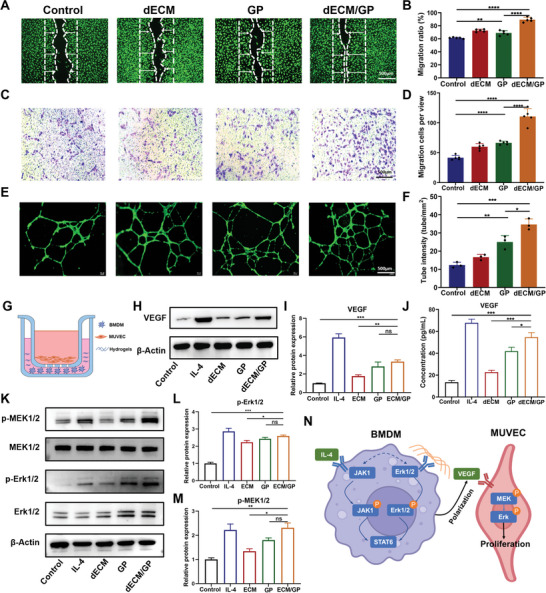
dECM/GP promoted migration, tube formation and proliferation of endothelial cells. A,B) Migration of HUVECs cocultured with medium or dECM, GP or dECM/GP hydrogels (*n* = 5). C,D) Representative images and quantification of transwell assay for HUVECs at 24 h (*n* = 5). E,F) Representative images and quantification of tube formation of HUVECs (*n* = 3). G) A crosstalk system was established by seeding BMDMs and hydrogels on the bottom and MUVECs on the inserted chamber. H) Western blot analysis of VEGF expression by treated BMDMs. I) Quantitative expression of VEGF (*n* = 3). J) ELISA of VEGF from supernate of BMDMs’ culture medium (*n* = 3). K) Western blot of p‐MEK1/2 and p‐Erk1/2. L,M) Quantitative expression of p‐MEK1/2 and p‐Erk1/2 (*n* = 3). N) Schematic diagram of dECM/GP for proliferation of endothelial cells.

Endothelial cell migration plays an important role in angiogenesis and vascular regeneration. dECM/GP hydrogel possessed the typical ECM feature with 3D nanofibrous and porous microstructure, which was expected to provide a favorable microenvironment for cell homing and proliferation. Next, wound scratch assay and transwell assay were performed to assess the effect of hydrogels on migration of human umbilical vein endothelial cells (HUVECs). For scratch assay, dECM/GP treatment could enhance the migration of HUVECs with faster closure of the cell‐free gap compared with the other groups (**Figure** **4**A,B). Moreover, the transwell assay also demonstrated that HUVECs incubated in dECM/GP hydrogels attracted more cells to migrate through the pores of chambers at 24 h (Figure 4C,D). These results demonstrated that the fibrous and porous microstructure of dECM/GP hydrogel could provide a favorable microenvironment for promoting cell migration, which had the potential to induce cell infiltration and promote endothelialization process for cardiac repair.

We further assessed the proangiogenetic effect of dECM/GP hydrogel in intro via tube formation assay. Following 12 h culture, the cells cocultured with dECM/GP hydrogel displayed denser tube intensities than that of dECM or GP hydrogels (Figure 4E,F), indicating that dECM/GP could facilitate vessel forming in the impaired tissue.

### 2.6 Proliferation of Endothelial Cells by dECM/GP Hydrogel via Crosstalk between Macrophages and Endothelial Cells

Endothelial cell proliferation is closely associated with neo‐angiogenesis and neo‐tissue regeneration. We first investigated whether dECM/GP hydrogel mediated proliferation of endothelial cells using CCK‐8 assay. After coculturing for 1, 3, and 5 days, the optical density increased over time, but did not show difference between the control and dECM/GP groups (Figure S8A, Supporting Information). The activation of Erk1/2, a key signal transduction factor in mitogen‐activated protein kinase (MAPK) pathway, also did not differ between the two groups (Figure S8B,C, Supporting Information). However, considering that dECM/GP hydrogel could promote M2 macrophage polarization and elevate VEGF mRNA expression (Figure 3D), we hypothesized that these immunomodulatory effects could result in endothelial cell proliferation.^[^ ^32^ ^]^ Therefore, we established a coculture system to investigate the crosstalk between macrophages and endothelial cells. As is shown in Figure 4G, macrophages derived from BMDMs were seeded in the bottom chamber, and subsequently were stimulated by hydrogels or IL‐4. And mouse umbilical vein endothelial cells (MUVECs) were seeded on the transwell insert chambers. After coculturing for 1 and 3 days, the cell viability of MUVECs was significantly improved in dECM/GP group compared with other groups determined by CCK‐8 assay (Figure S9, Supporting Information). The VEGF expression of macrophages was significantly elevated in GP and dECM/GP groups compared with control group (Figure 4H,I). Moreover, the concentration of VEGF in culture medium also upregulated, confirmed by enzyme linked immunosorbent assay (ELISA) (Figure 4J). These results verified that dECM/GP hydrogel promoted VEGF expression through activation of M2 phenotype macrophages. MEK1/2 is one of downstream factors of VEGF‐induced proliferation.^[^ ^33^ ^]^ Western blot analysis showed that phosphorylation of MEK1/2 and Erk1/2 of MUVECs were significantly upregulated by GP and dECM/GP treatment (Figure 4K–M), indicating that the proliferation of MUVECs were activated by VEGF‐MAPK pathway. Collectively, these data suggested that dECM/GP facilitated proliferation of endothelial cells via promoting M2 macrophage polarization and VEGF secretion, which indicated that the angiogenesis driven by dECM/GP might be accomplished by inflammatory regulation (Figure 4N).

### 2.7 Modulation of Cardiac Inflammation and Tissue Regeneration in a Rat MI Model by dECM/GP

First, in vitro degradation time of dECM/GP hydrogel was evaluated, which showed that dECM/GP degraded steadily for 21 days (Figure S10A, Supporting Information). Next, noninvasive fluorescence imaging was performed to investigate the in vivo degradation behavior of dECM/GP hydrogels after injecting into the back of mice. As is shown in Figure S10 (Supporting Information), the fluorescence intensity decreased gradually over time. dECM/GP hydrogels remained 49% on Day 7 and 12% on Day 14, indicating that dECM/GP hydrogels could degrade and take effect more than 14 days, which maintained significantly longer than dECM and GP hydrogels. Furtherly, we investigated the in‐situ degradation rate by injecting the dECM/GP hydrogel into the heart of rats. Figure S11 (Supporting Information) shows that dECM/GP maintained 33.2% of the fluorescence intensity on Day 14, fitting the time window of acute inflammatory response after myocardial infarction, which was conducive to construct a stable niche for cell regulation.

**Figure 5:**
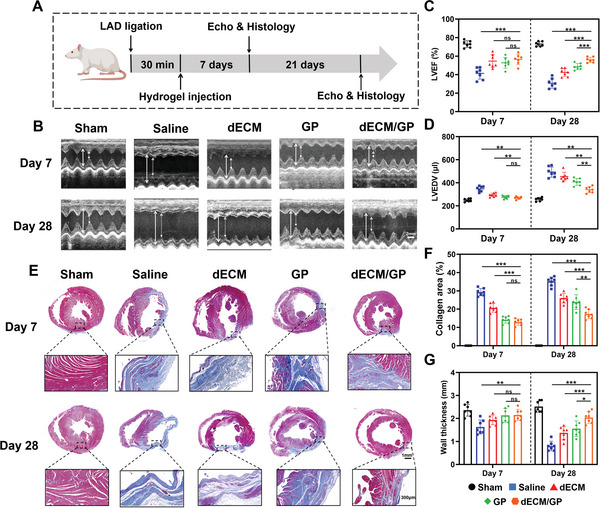
dECM/GP modulated cardiac repair in a rat MI model. A) Schematic illustration of treatment in a rat MI model. B) Representative images of echocardiography reflecting cardiac function. The white full line indicates the LVIDd; the white dotted line indicates the LVIDs. Quantification of LVEF C) and LVEDV D) by echocardiography (*n* = 7). E) Representative Masson trichrome staining of harvested heart at the level of papillary muscles. Quantification of collage area F) and wall thickness G) by Masson trichrome staining (*n* = 7).

Next the effects of dECM/GP hydrogel in cardiac tissue regeneration were verified in a rat acute MI model. **Figure** **5**A shows the key time point of the experiment procedure. Cardiac function was investigated by echocardiography. Results in Figure 5B–D demonstrated that after MI, cardiac function was markedly attenuated with decreased left ventricular ejection fraction (LVEF) and fractional shortening (LVFS) (Figure S12A,B, Supporting Information). The LVEF decreased from 73% (sham group) to 31% (saline group) on Day 28 (Figure S12A, Supporting Information), suggesting that MI led to the occurrence of heart failure with reduced ejection fraction (HFrEF). dECM/GP hydrogel improved the greatest values of LVEF and LVFS during the treatment. Interestingly, LVEF markedly decreased from Day 7 to Day 28 in saline (41.3%–30.7%), dECM (54.6%–42.4%) and GP (52.8%–48.6%) group (Figure S12A, Supporting Information). However, no significant drop was observed (56.4%–56.0%) in dECM/GP treatment and the value of LVEF maintained over 50% on Day 28 (Figure 5C; Figure S12A, Supporting Information), indicating that dECM/GP effectively reserved the contractility. Left ventricular dimension directly manifests the process of maladaptive remodeling.^[^ ^34^ ^]^ Figure 5D and Figure S12C (Supporting Information) show that left ventricular end‐diastolic volume (LVEDV) of saline treatment obviously enlarged on Day 28 and was 1.9‐fold to that of the sham group (492.9 µL vs 256.8 µL, *p* < 0.001), which was consistent with other echo indexes (LVIDd, LVIDs, and LVESV; Figure S13, Supporting Information), indicating the occurrence of dilated cardiomyopathy after MI. dECM and GP treatment obviously suppressed the ventricular expansion, while dECM/GP treatment further enhanced the effect (Figure 5D; Figures S12C and S13, Supporting Information). Geometrical changes are a major stimulus for LV remodeling illustrated by Law of Laplace, which reveals that ventricular wall stress is directly related to LV pressure and radius and is inversely proportional to twice the LV wall thickness.^[^ ^35^ ^]^ dECM/GP hydrogel significantly improved the LVEF, decreased the LV dimension and increased the wall thickness, thus adequately alleviating the wall stress and benefiting the LV remodeling, suggesting that dECM/GP hydrogel could effectively prevent the exacerbation of heart failure in the early stage after MI.

Histology was performed to investigate the morphological changes of post‐MI cardiac repair. Representative Masson trichrome staining showed that the permanent LAD ligation induced increased infarct size and attenuated wall thickness from Day 7 to Day 28 (Figure 5E). All the dECM, GP and dECM/GP treatment significantly reduced the collagen area and increased the wall thickness after injection (Figure 5F,G). The histological difference was not obvious between GP and dECM/GP group on Day 7. However, dECM/GP treatment further rescued more cardiomyocytes and better reserved wall thickness in the long term (Figure S14, Supporting Information). Moreover, widespread and intensive collagen deposition was observed in saline (collagen area: 35.1%), dECM (26.0%), and GP (23.9%) treatment on Day 28, indicating the pathological changes of heart failure, while dECM/GP (17.3%) inhibited this maladaptive healing process (Figure 5F).

**Figure 6:**
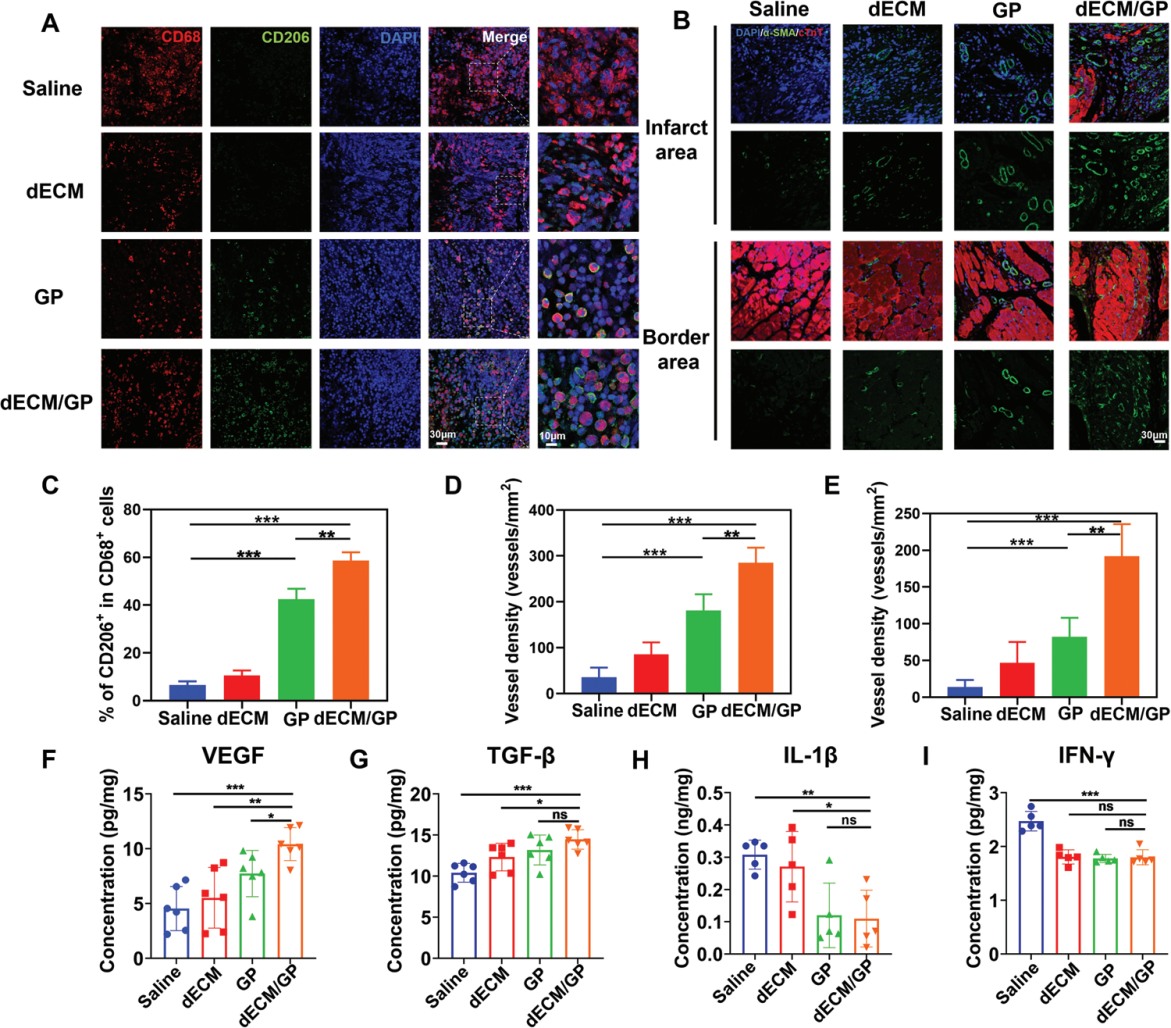
dECM/GP promoted M2 macrophage polarization and angiogenesis post MI. A) Representative pictures of CD206/CD68 immunofluorescence staining on Day 7 post MI. B) Representative pictures of *α*‐SMA immunofluorescence in infarct and border area on Day 28 post MI. C) Statistical data of the percentage of M2 macrophages (*n* = 5). Quantitative analysis of *α*‐SMA positive vessels in infarct area D) and border E) (*n* = 7). Concentrations of VEGF F), TGF‐*β* G), IL‐1*β* H), and IFN‐*γ* I) in infarct area determined by ELISA (*n* = 6 or *n* = 5).

In order to understand the mechanism of dECM/GP hydrogel in modulating the cardiac healing, we further evaluated the inflammatory response in infarct area. It is notable that at 7 days, the inflammation of infarct area was rampant and transmural in saline treatment, with the destruction of myocardial structure (Figure S15, Supporting Information). dECM/GP niche alleviated the inflammatory response compared with other groups, with less inflammatory cells infiltrating and more tissue generation. Furthermore, macrophages play a key role in post‐MI inflammatory and proliferative phases. We utilized immunofluorescence to elucidate the effect of macrophage polarization by dECM/GP hydrogel on cardiac tissue regeneration. **Figure** **6**A shows that abundant macrophages (CD68^+^) were mobilized and recruited in the infarct area after MI in all groups. GP and dECM/GP groups showed a greater number of CD206 positive (M2 macrophages) cells than dECM and control groups, demonstrating that more M2 type macrophages were induced by GP and dECM/GP niche. The percentage of M2 type in macrophages treated by dECM/GP (58.6%) was 5.6‐fold to that by dECM (10.5%) and 1.4‐fold to that by GP (42.4%) (Figure 6A,C). The results suggested that macrophages and monocytes were recruited and switched to M2 phenotype in the immunomodulatory niche, due to the high affinity of mannose receptors of GP and 3D nanofibrous microstructures in dECM/GP hydrogel.

Neo‐angiogenesis favors repair and repair of the infarcted myocardium. We have verified that dECM/GP could promote migration, tube formation and proliferation of endothelial cells in vitro, which are the key elements for vascular formation. Therefore, the proangiogenic bioactivity of dECM/GP hydrogel in vivo was testified. As is shown in Figure S16 (Supporting Information), more small vessels were observed in dECM/GP niche at the infarcted site. Neovascularization at 28 days was further evaluated by immunofluorescence staining of *α*‐smooth muscle actin (*α*‐SMA), a marker of functional arterioles. As is shown in Figure 6B,D,E, the number of *α*‐SMA^+^ blood vessels distributed at the infarct and border area in dECM/GP group. Furthermore, more cardiomyocytes (cTnT^+^) presented surrounding the neo‐vessels in the infarct area. These data confirmed that dECM/GP could facilitate angiogenesis and cardiomyocyte survival in vivo, which was mainly attributed to the promotion of enhancement of immune‐endothelial cell crosstalk. Next, the expression of representative proreparative cytokines including VEGF and transforming growth factor‐*β* (TGF‐*β*) and proinflammatory cytokines such as interleukin‐1*β* (IL‐1*β*) and interferon‐*γ* (IFN‐*γ*) in infarct area were detected by ELISA. As is shown in Figure 6F–I, the concentration of VEGF and TGF‐*β* were significantly upregulated by dECM/GP hydrogel treatment compared with other groups, while IL‐1*β* and IFN‐*γ* were markedly reduced. These above results suggested that dECM/GP hydrogel constructed an effective immunomodulatory niche in infarct size by altering the inflammatory cascade, regulating a proreparative immune response and promoting neo‐vessel formation.

### 2.8 Safety and Efficacy of dECM/GP for Treating a Swine MI Model

**Figure 7:**
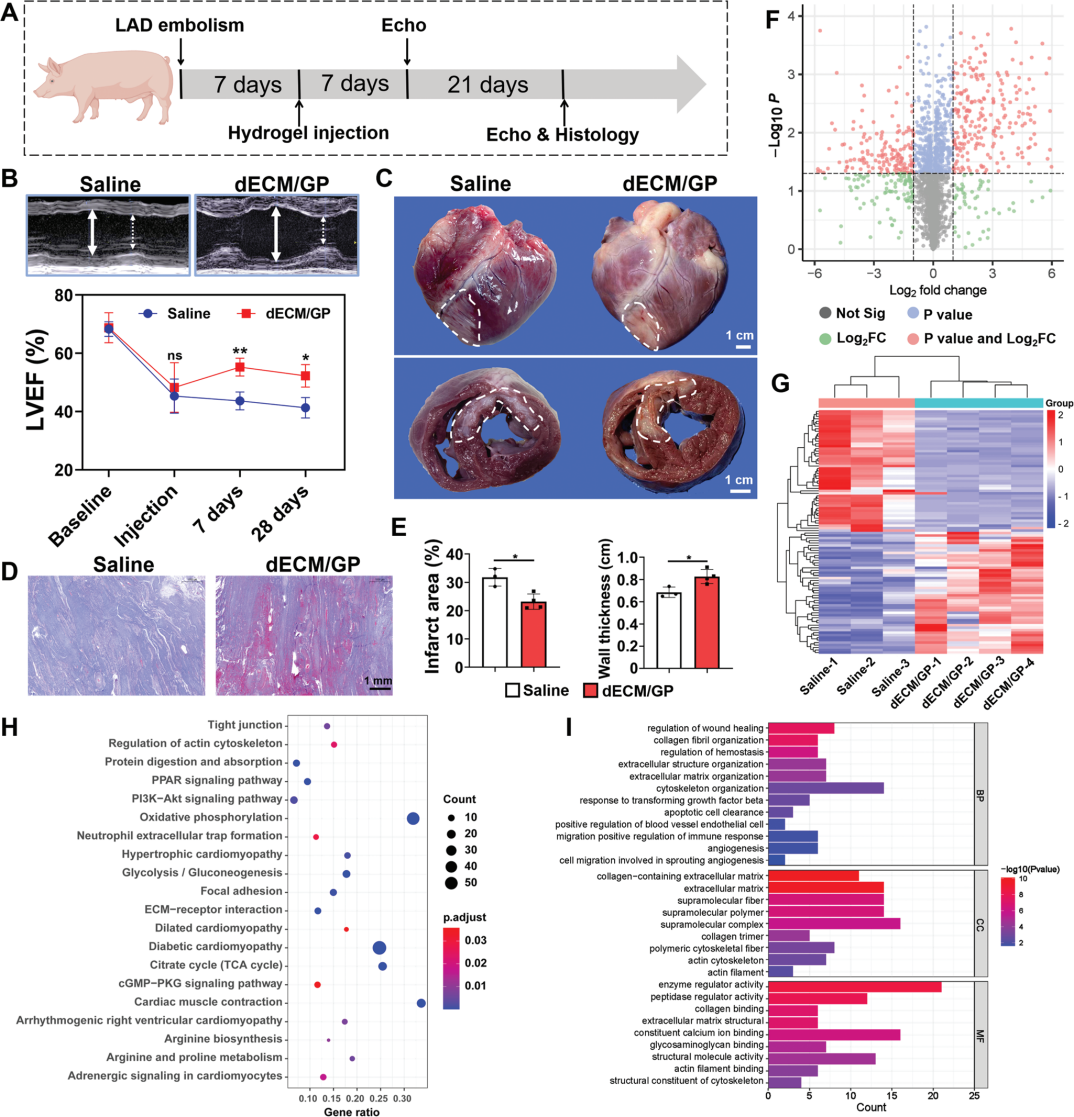
dECM/GP modulated cardiac repair in porcine MI model. A) Schematic illustration of treatment in a porcine MI model. B) Representative images of echocardiography reflecting cardiac function and quantification of LVEF. The white full line indicates the LVIDd; the white dotted line indicates the LVIDs. C) Gross examination of and cross section of harvested hearts on 28 days. D) Representative Masson trichrome staining of harvested heart in infarct area. E) Quantification of collage area and wall thickness (*n* = 3 or *n* = 4). F) Volcano plots. The red dots indicate the proteins selected based on adjusted P value < 0.05 and log_2_ FC > 2. G) Cluster heatmap of the top 50 upregulated and down regulated DEPs. H) KEGG pathway‐enrichment analysis of DEPs. I) GO pathway‐enrichment analysis of DEPs. BP indicates biological process; CC indicates cellular component; and MF indicates molecular function.

For clinical translation, we further evaluated the safety and effectiveness of dECM/GP hydrogel in a swine MI model (**Figure** **7**A). MI was induced by intracoronary embolization of mid left anterior descending coronary artery by spring coil (Figure S17, Supporting Information).^[^ ^36^ ^]^ Due to the high mortality of open‐chest surgery after MI, we randomly performed dECM/GP hydrogel or saline intramyocardial injection (*n* = 4) on Day 7 via a thoracotomy (Figure S18, Supporting Information).^[^ ^37^ ^]^ 28 days after injection, the swine were sacrificed to evaluate the effects. One pig in saline group died before injection and was excluded from the analysis. Echocardiography confirmed the successful modeling with LVEF decreased from 69% to 47% at 7 days post MI. After injection, dECM/GP treatment significantly improved the LVEF (55% vs 44%, *p* < 0.01) on Day 7 and maintained on Day 28 (52% vs 41%, *p* < 0.05) (Figure 7B). The ventricular dilation was also significantly mitigated by hydrogel treatment (Figure S19, Supporting Information). Figure 7C–E shows that the infarct area of the left ventricle and ventricular septum was significantly decreased to 23.2% in dECM/GP treatment vs 31.81% in saline group. Intriguingly, the infarct area of the LV free wall was significantly mitigated and the wall thickness was preserved in dECM/GP group compared with saline group (Figure 7E), while that of the ventricular septum remained no obvious different between the two groups (Figure 7B), probably because all the injection sites were distributed on the LV free wall. Masson trichrome stating confirmed the extensive fibrosis deposition in the saline group, which was significantly alleviated by dECM/GP treatment (Figure 7C). Furthermore, more myocardium was observed in infarct area, contributing to the greater cardiac function. The histology of other important organs (e.g., lung, kidney, liver, and spleen) and routine blood tests of dECM/GP treated swine were comparable to those of saline group, confirming that the safety of this hydrogel (Figure S20 and Table S2, Supporting Information).

We further performed the proteomics to investigate the mechanisms of cardiac tissue regeneration by dECM/GP hydrogel. A total of 2868 proteins were identified, with 481 differentially expressed proteins (DEPs) including 291 proteins upregulated and 190 proteins downregulated. The DEPs were visualized by volcano plots (Figure 7F), and the top 50 most significantly up and down regulated DEPs were shown in the cluster heatmap (Figure 7G), indicating the obvious changes of protein expression caused by dECM/GP hydrogel. KEGG and GO pathway‐enrichment analyses were conducted to demonstrate the main biological processes regulated by dECM/GP hydrogel. Significantly enriched terms in KEGG included contractibility and heart failure pathways such as adrenergic signaling in cardiomyocytes, cardiac muscle contraction, regulation of actin cytoskeleton, dilated cardiomyopathy, hypertrophic cardiomyopathy and diabetic cardiomyopathy, demonstrating that the dECM/GP hydrogel improved the cardiomyocyte vitality and function (Figure 7H). In addition, pathways involving in host cell interactions with ECM such as ECM‐receptor interaction, focal adhesion and tight junction were significantly upregulated, indicating the homing biofunction of the niche. Metabolic pathways related to mitochondrial function and glycometabolism such as oxidative phosphorylation, citrate cycle (TCA cycle) and glycolysis/gluconeogenesis were enhanced, which could contribute to the improvement of cardiomyocyte oxygen consumption and contraction, consistent with recent researches demonstrating that proteins from dECM could provoke cardiomyocyte regeneration.^[^ ^38^ ^]^ GO enrichment further confirmed that the DEPs were mainly associated with wound healing, ECM organization, positive regulation of endothelial cell migration, regulation of immune response, and angiogenesis, which conformed to the “HELP” regenerative strategy (Figure 7I). All these data demonstrated dECM/GP immunomodulatory niche could reconstruct the cardiac ECM, promote heart healing, and improve cardiomyocyte viability after MI.

## 3 Discussion

Hydrogels have been extensively investigated for heart repair in preclinical studies.^[^ ^10^, ^39^ ^]^ Representatively, the clinical trials have demonstrated great advancement and biosafety of polysaccharide or dECM based hydrogels.^[^ ^11^, ^13^, ^40^ ^]^ dECM hydrogel possesses native ECM structure and ingredients, but is limited by its fragile and weak characterization with rapid degradation rate, which would not be expected to act as a mechanical support.^[^ ^13^ ^]^ Moreover, dECM lacks of sufficient bioactivities for promoting tissue regeneration. Matrix metalloprotease released by M1 type macrophages after acute MI may mitigate the effect of ECM reconstruction.^[^ ^15c^ ^]^ On the contrary, polysaccharide with better mechanical stability and self‐healing property could provide mechanical support to mitigate the stress injuries of myocardial wall,^[^ ^41^ ^]^ but the dense network of the hydrogel could hinder the infiltration and proliferation of the healing‐related cells (Figure 2B). Besides, the bioactivities of polysaccharide hydrogel in myocardial regeneration were also rarely explored.^[^ ^11^, ^13^ ^]^ To circumvent these limitations, drugs,^[^ ^42^ ^]^ cytokines,^[^ ^43^ ^]^ or stem cell delivery^[^ ^44^ ^]^ have been encapsulated in hydrogels to confer extra biofunctions such as immunoregulation, angiogenesis or metabolism modulation for improving myocardial regeneration. However, they were impeded by sophisticated fabrication and storage, uncontrolled drug release, high cost, or drug‐related side effects. In this study, the biophysical and biochemical cues of dECM/GP were delicately designed to regulate the host cell response and tissue repair.^[^ ^8^ ^]^ Biophysical characteristics, such as mechanical properties, microporosity, duration of degradation influence cell infiltration and differentiation.^[^ ^45^ ^]^ dECM/GP possesses a stiffness of ≈1000 Pa, which not only renders machinal strength to alleviate the stress injuries of myocardial wall and promote myocardial remodeling.^[^ ^26^ ^]^ The microporous structure (≈3 µm) of dECM/GP similar to native cardiac ECM could favor cellular infiltration and angiogenesis for improving the vascularization and tissue ingrowth. Moreover, dECM/GP hydrogel contains abundant proteoglycans and glycoproteins further facilitate the adhesion and migration of healing‐related cells.^[^ ^46^ ^]^ Furthermore, the introduction of mannose unit ad KK peptide could precisely regulate the macrophage polarization by modulating extracellular signaling. As a result, the integral structure of dECM/GP hydrogels can sequentially and synergistically regulate cell recruitment, differentiation and proliferation, thus boosting the native repair potential without the presence of cytokines, drugs or stem cells. The simple and economical manufacture process and storing condition (4 °C before use) of dECM/GP hydrogel would also be favored for clinical use.

Based on the present results, our study still remained some limitations for the future work. Firstly, the dECM/GP was prepared by porcine heart tissue. However, the immunogenicity and potential virus may limit its application to treat human disease. The advancement of genetically modified porcine heart may herald the beginning of an era of heterologous tissue implantation.^[^ ^47^ ^]^ Secondly, future investigation could focus on the mechanisms of dECM/GP modulating cardiomyocytes and fibroblasts to comprehensively map the involved cell populations and signaling pathways. Thirdly, feasibility of transcatheter injection of dECM/GP hydrogel rather than open‐chest access can be further explored, which would reduce the surgical trauma and improve the therapeutic effect.^[^ ^12^, ^13^ ^]^

## 4 Conclusion

Here, we successfully developed a “HELP” regenerative strategy‐based dECM/GP hybrid hydrogel as an immunomodulatory niche for cardiac tissue repair post MI. For host cell homing, dECM/GP hydrogel possess a native structure and bioactive ingredients derived from porcine cardiac tissue, allowing for cell adhesion and migration. Furthermore, the introduction of GP into the system by click chemistry conferred stronger mechanical stability and rapid self‐healing ability suitable for cardiac implantation. For elaborating cell differentiation, the mannose unit and KK peptide of dECM/GP hydrogel could specifically polarize more macrophages toward M2 type through JAK1 and Erk1/2 pathway and regulate post‐MI inflammatory cascade. For proliferating, recruited endothelial cell could proliferate and form neo‐vessels in the niche via immune‐endothelial crosstalk, which positively activated the resident cell to orchestrate tissue regeneration. As a result, dECM/GP hydrogel could construct an immunomodulatory niche conforming to “HELP” regenerative strategy, which effectively improve cardiac repair by attracting host cell infiltration, controlling macrophage differentiation and promoting endotheliocyte proliferation, without the presence of cytokines, drugs or stem cells. We envision that dECM/GP hydrogel with simple and economical manufacture process could provide clinical opportunity to effectively improve the prognosis for MI patients.

## 5 Experimental Section

### Materials

All animal experiments were approved by Institutional Animal Care and Use Committee, Fuwai hospital, Chinese Academy of Medical Sciences and Center of Tianjin Animal Experiment Ethics Committee and Authority for Animal Protection (FW‐2022‐0021). GM (*η* ≥ 15 000 mPa s) was purchased from Yuanye Bio‐Technology Co., Ltd (Shanghai, China). KK peptide was purchased from Bankpeptide biological technology CO., Ltd (Hefei, China). 4‐Maleimidobutyric acid, 1‐ethyl‐3‐(3‐dimethylaminopropyl) carbodiimide (EDC), and 4‐dimethylaminopyridine (DMAP) were purchased from Aladdin Chemical Reagents Co., Ltd. (Shanghai, China).

### Preparation of dECM/GP Hydrogel

To fabricate dECM hydrogel, porcine hearts were harvested, decellularized, lyophilized and solubilized under sterile conditions following a modified protocol reported previously.^[^ ^16^ ^]^ Briefly, the porcine ventricular tissue was cut into pieces of 2 mm in thickness, rinsed with deionized water, and decellularized by 1% (w/v) sodium dodecyl sulfate (SDS) for 4–5 days, until the tissue was bleached and transparent. The tissue was then stirred in deionized water for another 2 days to remove the detergents. The decellularized matrix was stained with Masson trichrome staining to confirm the absence of cells. Subsequently, the decellularized tissue was frozen (−20 °C), lyophilized, and milled into powder using Wiley Mini Mill. Then, the matrix powder was dissolved in 0.1 m HCl and digested with pepsin (Sigma, p6887) with mass ratio of 1:10 (pepsin:matrix). After being incubated for 24 h, the pH of the aqueous solution was adjusted to 7.5 by adding 1 m NaOH and diluted with 1 × PBS. The final concentration of decellularized heart matrix ranged from 10 to 60 mg mL^−1^.

The MAL‐GM, was synthesized by esterification. Briefly, GM (2 g, 2% w/v) was dissolved in double distilled water, and the 4‐maleimidobutyric acid (2.2 g) in aqueous solution was added dropwise to the reaction mixture, with EDC (7 g) and DMAP (0.5 g) as catalysts. Then the mixture was stirred overnight at room temperature. After being dialyzed against water for 5 days, the product of MAL‐GM was lyophilized, and kept under nitrogen before use. The synthesis of MAL‐GM was determined by ^1^H‐NMR and UV–vis spectrum.

dECM/GP hydrogel was prepared through conjugating the ECM and KK peptide on the backbone of GM by simply mixing the dECM (50 mg mL^−1^), MAL‐GM (40 mg mL^−1^) and KK peptide (20 mg mL^−1^) at the volume ratio of 1:1:1. The GP was formed by mixing the MAL‐GM (40 mg mL^−1^) and KK peptide (20 mg mL^−1^) in solution at the volume ratio of 1:1. Details of SEM, rheology and CD spectrum tests are provided in supplementary materials.

### Wound scratch, Transwell, and Tube Formation Assay

For wound scratch assay, 1.5 × 10^5^ HUVECs were seeded in a 6‐well plate and incubated to reach confluence. The wound was scratched using a 1 mL pipette tip and washed with PBS to remove detached cells. Then the cells were cultured in medium supplemented with 20% (v/v) hydrogels. At 12 h postwounding, HUVECs were stained with calcein (0.1%) for 30 min and photographed.

For transwell assay, 1 × 10^4^ HUVECs were seeded in the upper chamber of a 24‐well plate. The lower chamber was loaded 400 µL cell culture medium with 100 µL hydrogels. After incubation for 24 h, the migrated cells on the lower chamber were stained with crystal violet solution (0.5%) for 30 min and were counted.

For tube formation assay, 50 µL Matrigel (BD, 356234) was added into a precooled 96‐well plate and incubated for 30 min at 37 °C. After that, 40 µL cell culture medium mixed with 10 µL hydrogels containing 5 × 10^3^ HUVECs were seeded and further incubated for 12 h. HUVECs were stained with calcein solution and the tube formation density was quantified using ImageJ software.

### Macrophage Polarization

BMDMs were isolated from C57BL/6 mice (6 weeks old, Vital River Laboratory, China). After lysing red blood cells, the collected cells were seeded in 6 well plates and cultured with RPMI 1640 medium supplemented with 10% heat‐inactivated fetal bovine serum and 20 ng mL^−1^ M‐CSF (MCE, HY‐P7085). After incubation for 6 days, adhered cells were BMDMs. BMDMs were cocultured with IL‐4 (40 ng mL^−1^, PeproTech, 214‐14) or hydrogels for 2 days. After that, treated cells were seeded in confocal dishes, stained with FITC‐labeled phalloidine (Solarbio, CA1620), APC‐labeled CD206 (Biolegend, 141708) and DAPI (Solarbio, C0065) according to the manufacture's guidelines and were observed by confocal laser scanning microscopy (TCS SP5II, Leica, Ernst‐Leitz‐Strasse, Germany). BMDMs were also stained with FITC‐labeled anti‐CD86 antibodies (Biolegend, 105006), PE‐labeled F4/80 antibodies (Biolegend, 123110) and APC‐labeled CD206 (Biolegend, 141708) and then were analyzed by flow cytometry (C6, BD, USA). Procedures of RT‐PCR, western blot and ELISA can be found in supplementary materials.

### Crosstalk System

BMDMs stimulated by M‐CSF for 6 days were seeded in 6‐well plates with a density of 2 × 10^5^ per well. The culture medium was supplied with 20% (v/v) hydrogels.1.5 × 10^5^ MUVECs were seeded on the inserted chamber with a pore size of 0.4 µm (Corning, 3450). After coculturing for 2 days, the cells were collected for further analysis.

### In Vivo Fluorescence Imaging

A total of 100 µL hydrogels coupled with rhodamine were subcutaneously injected into the back of mice. The fluorescence intensity was quantified at schedule time using the Maestro imaging system (CRI, USA).

### Rat MI Model

Male Sprague–Dawley (SD) rats (Vital River Laboratory, China) weighting 180–200 g (6 weeks) were used for building MI models. Acute MI was performed as previously described.^[^ ^41^ ^]^ Briefly, rats were anesthetized using inhaled isoflurane. After that, endotracheal intubation was performed with 16G trocar, and breathing was maintained by a ventilator. The intercostal muscles were separated at the third or fourth intercostal space until the heart was exposed. Then the rats were randomly assigned to 5 groups: sham, saline, dECM, GP, dECM/GP (*n* = 14 in each group). The left anterior descending coronary artery was permanently ligated with an 6‐0 nylon suture. After 30 min of ischemia, a total of 100 µL saline or hydrogels were injected at 4 evenly distributed border sites of the infarction area. The sham group were subjected to the same procedure without ligation and injection. The chest was carefully closed and the penicillin was injected with for prevention of infection. Details of echocardiographic measurement, histology and ELISA assay are provided in supplementary materials.

### Swine MI Model

Pigs weighing 25–30 kg were used in this study. After anesthesia, an acute MI was produced by spring coil embolism in the mid‐left anterior descending (LAD) coronary artery.^[^ ^36^ ^]^ Echocardiography, blood tests and electrocardiography were monitored throughout the procedure. On Day 7, an open‐chest procedure was performed for injection.^[^ ^37^ ^]^ Pigs received intramyocardial injections of 2 mL of either dECM/GP hydrogel or saline. Four or five injection sites were evenly distributed along the infarct area of the LV free wall. Echocardiography was monitored at 1, 7, and 30 days after injection. The pigs were sacrificed on 28 days. The harvested heart was analyzed by histology and the tissue in the infarct area was analyzed by proteomics (see the supplementary materials).

### Statistical Analysis

Statistical analysis was performed using GraphPad Prism 8 (GraphPad Software) and data were expressed as mean ± SD. Comparisons between two groups were performed with unpaired Student's *t*‐test, while for multiple group comparison, one‐way ANOVA was used with Bonferroni post correction. Statistical significance is denoted by *p < 0.05, **p < 0.01, and ***p < 0.001.

## Conflict of Interest

The authors declare no conflict of interest.

## Author Contributions

P.K. and J.D. equally contributed to this work. Conceptualization: Z.F., W.W., X.P., P.K.; Methodology: Z.F., W.W., P.K., W.L., Z.L., X.L., J.W., Q.S., W.B.; Investigation: P.K., J.D., S.W., W.O., F.Z., S.F., Y.X.; Visualization: PK, HY, ZF, W.W., D.Z.; Funding acquisition: W.W., X.P.; Project administration: P.K., J.D., L.W., G.Z., F.Z., S.W.; Supervision: W.W., X.P., X.L., Z.F., R.G.; Writing—original draft: P.K., Z.F., J.D., W.W.; Writing—review and editing: P.K., J.D., W.L., Z.L., R.G., X.L., J.W., Q.S., B.W., W.O., S.W., F.Z., S.F., D.Z., Y.X., G.Z., H.Y., Z.F., W.W., X.P.

## Supporting information

Supporting Information

## Data Availability

The data that support the findings of this study are available from the corresponding author upon reasonable request.
