# Supplementary material for: Extracellular Matrix/Glycopeptide Hybrid Hydrogel as an Immunomodulatory Niche for Endogenous Cardiac Repair after Myocardial Infarction
Source: Adv Sci (Weinh). 2023 Jun 15;10(23):2301244. doi: 10.1002/advs.202301244 (PMC10427380; doi:10.1002/advs.202301244)
Supplement: Supplementary file 1 — Supporting Information [file ADVS-10-2301244-s001.pdf]

ADVANCED SCIENCE
Open Access

# Supporting Information

for *Adv. Sci.*, DOI 10.1002/advs.202301244

Extracellular Matrix/Glycopeptide Hybrid Hydrogel as an Immunomodulatory Niche for Endogenous Cardiac Repair after Myocardial Infarction

*Pengxu Kong, Jing Dong, Wenchao Li, Zefu Li, Rui Gao, Xiang Liu, Jingrong Wang, Qi Su, Bin Wen, Wenbin Ouyang, Shouzheng Wang, Fengwen Zhang, Shuyi Feng, Donglin Zhuang, Yongquan Xie, Guangzhi Zhao, Hang Yi, Zujian Feng*, Weiwei Wang* and Xiangbin Pan**

Supporting Information

**Extracellular Matrix/Glycopeptide Hybrid Hydrogel as an Immunomodulatory Niche for Endogenous Cardiac Repair after Myocardial Infarction**

*Pengxu Kong, Jing Dong, Wenchao Li, Zefu Li, Rui Gao, Xiang Liu, Jingrong Wang, Qi Su, Bin Wen, Wenbin Ouyang, Shouzheng Wang, Fengwen Zhang, Shuyi Feng, Donglin Zhuang, Yongquan Xie, Guangzhi Zhao, Hang Yi, Zujian Feng*, Weiwei Wang* and Xiangbin Pan**

**This file includes:**

Methods

Figures S1 to S20

Tables S1-S2

## Methods

### SEM, rheology and CD spectrum

The interior morphology of decellularized cardiac tissue, dECM, GP, and dECM/GP hydrogels was investigated by SEM (S-4800, Hitachi, Japan). Samples were fixed in 2.5% glutaraldehyde, dehydrated by $CO_2$ critical point drying (K850X, Emitech), quick-frozen in liquid nitrogen, lyophilized and then coated with gold particles. Three random areas of each sample by SEM were captured for analyzing the morphology of the hydrogels.

Rheological properties of dECM hydrogel, GP hydrogel, and dECM/GP hydrogel were evaluated by an AR 2000ex rheometer (TA). $G'$ and $G''$ were measured over a frequency sweep from 1 to 100 rad/s at a strain of 1%, a time sweep of 500 s with 1% strain and 1 rad/s frequency, and a strain sweep from 1 to 100% with 1 rad/s frequency at 37 °C. The self-healing properties were investigated by a continuous strain sweep with an alternative large oscillation force (55%) and a small one (2%).

CD spectrum was measured using a CD instrument (J-810, JASCO, Japan). Hydrogels were diluted into aqueous solutions (0.1 mg/mL). The UV region was scanned between 190 and 260 nm at 1 nm intervals and 3 s average time, using a 0.1 cm path length quarts cuvette. The secondary structure prediction was analyzed by Bestsel (https://bestsel.elte.hu/index.php).

### CCK-8 assay and Live/Dead staining

The biocompatibility of hydrogels was tested by Cell Counting Kit-8 (CCK-8) assay (Dojindo, CK04). Briefly, L929 and H9C2 cells were seeded at a density of $5\times10^3$ cells/well in 96-cell plates for 24 h. After that, the culture medium was replaced by 80 μL fresh medium

mixed with 20 μL hydrogels, and the cells were cultured for another 3 days. The culture medium was replaced by new medium containing 10% CCK-8 kit (v/v) and cells were cultured for another 1 h. The absorbance was measured at the wavelength of 450 nm.

Live/Dead staining was performed using Calcein/PI Cytotoxicity Assay Kit (Beyotime, C2015M). L929 and H9C2 cells were seeded at a density of $1\times10^5$ cells/well in 6-cell plates and were co-cultured with dECM/GP hydrogel for 1 and 3 days. The cells were stained with calcein and PI and observed under fluorescence microscope.

**In vitro blood compatibility**

The hemolysis assay and blood coagulation assay were conducted to evaluate the blood compatibility of the ECM/GP hydrogel. For hemolysis assay, the fresh rat blood with anticoagulation was centrifuged at 1500 rpm for 15 min to acquire the lower blood cells, and then the blood cells were diluted to 5 v/v% by saline. The samples of hydrogels were immersed in the diluted blood cells at a volume ratio of 10% at 37℃ for 1 h. After centrifugation at 3000 rpm for 5 min, the supernatant was collected for absorbance measurement at 540 nm by an enzyme standard instrument. Sterilized deionized water and saline were used as positive and negative control, respectively. Hemolysis rate was calculated as follows: Hemolysis rate (%) = $(A_s-A_n)/(A_p-A_n)\times100\%$, where $A_s$, $A_p$ and $A_n$ represent the absorbance of samples, positive control and negative control, respectively.

For blood coagulation assay,[1] 50 μL dECM/GP hydrogel was firstly deposited on a 96 well plate. A total of 100 μL of 0.1 M calcium chloride ($CaCl_2$) was added into 900 μL of citrated blood from rats and vortexed for 10 s. Then, 50 μL of the blood was deposited on the hydrogel. After incubation at 37°C at the schedule time, the uncoagulated red blood cells

were hemolyzed with 100 μL deionized water. 50 μL of the supernatant was collected and diluted with 450 μL saline for absorbance measurement at 540 nm. The 96 well without hydrogel was set as a blank control and 50 μL of citrated blood diluted in 450 μL deionized water was utilized as the negative control. The blood colt index (BCI) was calculated as follows: BCI = $A_s/A_n$×100%, where $A_s$ and $A_n$ represent the absorbance of samples and negative control, respectively.

**RT-PCR**

RNA was extracted from treated BMDMs by using TRIzol reagent. cDNA was synthesized by using GoScript Reverse Transcription System (Promega, A5001). For real-time PCR analysis, 20 ng of input RNA was amplified according to a pre-designed procedure in an IQ5 detection system (Bio-Rad, USA) using SYBR Green Master Mix (Promega, A6001) and 500 nmol/L of gene-specific primers. Relative expression was evaluated by the comparative Ct (threshold cycle) method and normalized to the gene expression of β-actin. The forward and reverse primer sequences were listed in Table S1.

**Western Blot**

Cell lysates were lysed by RIPA lysis buffer (Beyotime, P0013B) containing 1 mM PMSF (Beyotime, ST506). After incubation for 30 min on ice, the supernatant was collected after centrifugation. Then protein was denatured at 95 °C for 10 min. After mixing with loading buffer containing bromophenol blue, protein samples were separated by a 10% sodium dodecyl sulfate polyacrylamide gel 4hosphor4oresis (SDS-PAGE) and transferred onto poly (vinylidene difluoride) (PVDF) membranes (0.45 μm). The membranes were blocked by bovine albumin and then incubated with antibody over night at 4°C. After washing with

TBST, the membranes were incubated with horseradish peroxidase-conjugated secondary antibodies for 1 h at room temperature. The proteins on the membranes were visualized by Chemiluminescence Imaging system (ChemiScope 6000 Pro, China). The intensity of immunoreactive bands was quantified by ImageJ software. The primary antibody included 5hosphor-MEK1/2 (Ser217/221) (1:1000, Cell Signaling, #9154), 5hosphor-p44/42 MAPK (Erk1/2) (Thr202/Tyr204) (1:1000, Cell Signaling, #4370), MEK1/2 (1:1000, Cell Signaling, # 4694), p44/42 MAPK (Erk1/2) (1:1000, Cell Signaling, # 4695), JAK1 (1:1000, Abcam, ab133666), 5hosphor-JAK1 (1:1000, Abcam, ab138005), VEGF (1:1000, Abcam, ab214424), STAT6 (1:1000, Abcam, ab32520) and β-actin (1:2000, Abcam, ab8226). The secondary antibody included HRP-labeled goat anti-mouse IgG (1:2000, Beyotime, A0216) and HRP-labeled goat anti-rabbit IgG (1:2000, Beyotime, A0208).

**ELISA**

The supernatant in the bottom chamber of the crosstalk system was collected for VEGF concentration detection using a Mouse VEGF ELISA Kit (Beyotime, PV957) according to the manufacturers' instructions.

**In vitro degradation**

To investigate the in vitro degradation time, 500 μL dECM/GP hydrogel was immersed in PBS at 37 °C shaking with 60 rpm. The supernatant was removed after incubation for 3, 7, 14, 21 days, and the hydrogel was freeze-dried and weighed.[2] The weight remaining ratio was calculated as follows: weight remaining ratio = $W_n$ / $W_0$ × 100%, where $W_0$ represents the dried weight of dECM/GP hydrogel and $W_n$ represents the weight of day n.

**In vivo degradation in heart**

A total of 100 μL dECM/GP hydrogel labelled with Cy5 maleimide was injected into the rat heart. The heart was harvested at the scheduled time, and the fluorescence images was recorded by Maestro imaging system (CRI, USA) under the absorbance at 605 nm.

**Echocardiographic measurement**

On Day 7 and 28 after surgery, cardiac function was determined by echocardiography (VisualSonics, Vevo 2100). At the level of papillary muscles, parasternal long and short axis was used to measure left ventricular internal diameter end diastole (LVIDd) and end systole (LVIDs), and thus calculate the LVEF, LVFS, LVEDD and Left Ventricular End Systolic Diameter (LVESD).

**Histology**

On Day 7 and 28, rats were sacrificed and the hearts were harvested (n = 7 in each group at each time point), fixed with 4% paraformaldehyde, embedded in paraffin, and sliced into 4 μm thick sections. Then the sections were stained for HE, Masson trichrome and immunofluorescence. For macrophage polarization investigation, the sections were incubated with the primary antibody of rabbit anti-CD68 (1:200, Abcam, ab125212), mouse anti-CD206 (1:200, Proteintech, 60143-1-Ig), mouse anti-α-SMA (1:200, Abcam, ab7817), and rabbit anti-cardiac troponin T (1:200, Abcam, ab209813). After incubation overnight, sections were incubated with Alexa Flour 488 donkey anti-mouse IgG (1:200, Thermo Fisher Scientific) and Alexa Flour 594 donkey anti-rabbit IgG (1:200, Thermo Fisher Scientific) corresponding to the primary antibody. Images were observed by CLSM and quantitatively analyzed by ImageJ software.

**ELISA assay for heart tissue**

The concentration of cytokines in rat heart tissue including VEGF, TGF-β1, IL-1β and IFN-γ were analyzed by ELISA. Briefly, 15 mg heart tissue were lysed by 500 μl RIPA lysis buffer containing 1 mM PMSF. The supertanent was collected for concentration detection using Rat VEGF ELISA Kit (CUSABIO, CSB-E04757r), TGF-β1 ELISA Kit (CUSABIO, CSB-E04727r), IL-1β ELISA Kit (CUSABIO, CSB-E08055r) and IFN-γ ELISA Kit (CUSABIO, CSB-E04579r) according to the manufacturers' instructions.

**Proteomics**

**Protein extraction:** 300 μL of 8M urea was added to the sample and the protease inhibitor was added at 10% of the lysate. After centrifuging at 14,100×g for 20 min, the supernatant was collected. The protein concentration was determined using Bradford method, rest was frozen to 80°C.

**Protein digestion and desalination:** 100 μg aliquot of extracted proteins from each sample was then subjected to reduction. 200 mM dithiothreitol (DTT) solution was added and incubated at 37 °C for 1 h. The sample was diluted 4 times by adding 25 mM ammonium bicarbonate (ABC) buffer, then trypsin (trypsin: protein =1:50) was added and incubated at 37°C overnight. 50μL 0.1% FA was added to terminate the digestion over 24 hours. 100μl 100% ACN was used to wash the C18 column at 1200rpm for 3min. The column was washed once with 100 μl of 0.1% FA at 1200 rpm for 3 min. The EP tube was replaced and the sample was added and centrifuged at 1200rpm for 3min. The column was washed twice with 100 μl of 0.1% FA at 1200 rpm for 3 min, then washed once with 100 μl of pH 10 water. The EP tube was replaced and eluted with 70% ACN. The eluents of each sample were combined and lyophilized, stored at 80°C until loading.

**LC MS/MS Analysis:** Nanoflow LC MS/MS analysis of tryptic peptides was conducted on a quadrupole Orbitrap mass spectrometer (Q Exactive HF X, Thermo Fisher Scientific, Bremen, Germany) coupled to an EASY nLC 1200 ultra high-pressure system (Thermo Fisher Scientific) via a nano electrospray ion source. 500 ng of peptides were loaded on a 25 cm column (150 μm inner diameter), packed using ReproSil Pur C18 AQ 1.9 μm silica beads. Peptides were separated using a gradient from 8 to 12% B in 5 min, then 12% to 30 % B in 33 min and stepped up to 40% in 7 min followed by a 15 min wash at 95% B at 600 nl per minute where solvent A was 0.1% formic acid in water and solvent B was 80% ACN and 0.1% formic acid in water. The total duration of the run was 60 min. Column temperature was kept at 60 °C using an in house developed oven. Briefly, the mass spectrometer was operated in "top 40" data dependent mode, collecting MS spectra in the Orbitrap mass analyzer (120,000 resolution, 350 1500 m/z range) with an automatic gain control (AGC) target of 3E6 and a maximum ion injection time of 80 ms. The most intense ions from the full scan were isolated with an isolation width of 1.6 m/z. Following higher energy collisional dissociation (HCD) with a normalized collision energy (NCE) of 27, MS/MS spectra were collected in the Orbitrap (15,000 resolution) with an AGC target of 5E4 and a maximum ion injection time of 45 ms. Precursor dynamic exclusion was enabled with a duration of 16 s.

**Data analysis**

The identification and quantitation of protein: All RAW files were analyzed using the Proteome Discoverer suite (version 2.4, Thermo Fisher Scientific). MS2 spectra were searched against the UniProtKB human proteome database containing both Swiss--Prot and TrEMBL human reference protein sequences (90,411 target sequences downloaded on 21

April 2015). The Sequest HT search engine was used, and parameters were specified as follows: fully tryptic specificity, maximum of two missed cleavages, minimum peptide length of 6, fixed carbamidomethylation of cysteine residues (+57.02146Da), variable modifications for oxidation of methionine residues (+15.99492Da), precursor mass tolerance of 15 ppm and a fragment mass tolerance of 0.02Da for MS2 spectra collected in the Orbitrap. Percolator was used to filter peptide spectral matches and peptides to a false discovery rate (FDR) of less than 1%. After spectral assignment, peptides were assembled into proteins and were further filtered based on the combined probabilities of their constituent peptides to a final FDR of 1%. As default, the top matching protein or 'master protein' is the protein with the largest number of unique peptides and with the smallest value in the percent peptide coverage (that is, the longest protein). Only unique and razor (that is, parsimonious) peptides were considered for quantification.

The functional analysis of protein and DEP Gene Ontology (GO) were conducted using the interproscan--5 program against the non-redundant protein database, and the databases KEGG (Kyoto Encyclopedia of Genes and Genomes) were used to analyze the protein family and pathway.

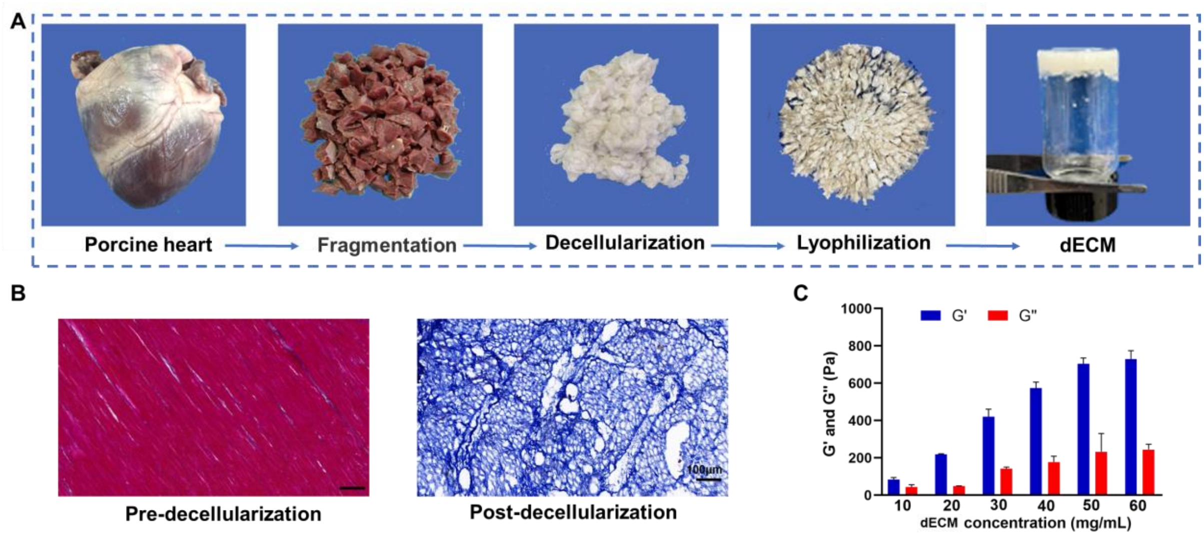


**Figure S1. Preparation and characterization of dECM hydrogel. (A)** Manufacturing procedures of decellularized porcine cardiac dECM hydrogels. **(B)** Masson trichrome staining of cardiac tissue before and post decellularization. **(C)** The influence of dECM concentration on rheology.

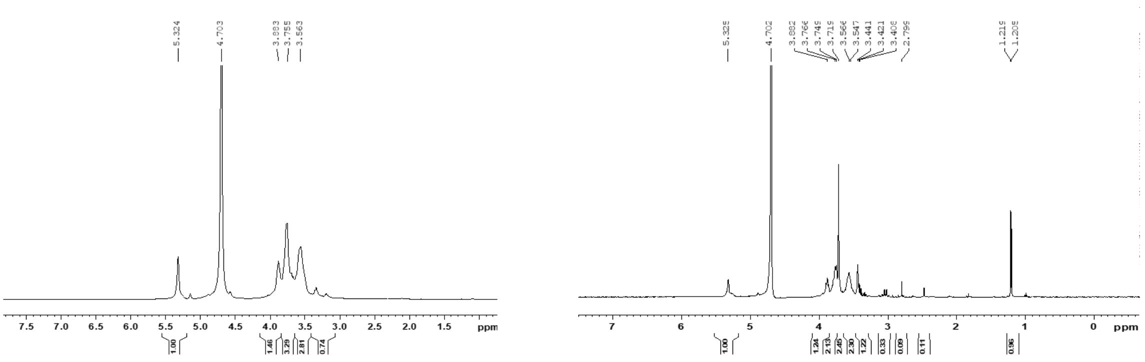

**GM** **MAL-GM**

**Figure S2. H-NMR of MAL-GM.** The formation of MAL-GM was confirmed by an extra peak (double bond) at 1.2 parts per million.

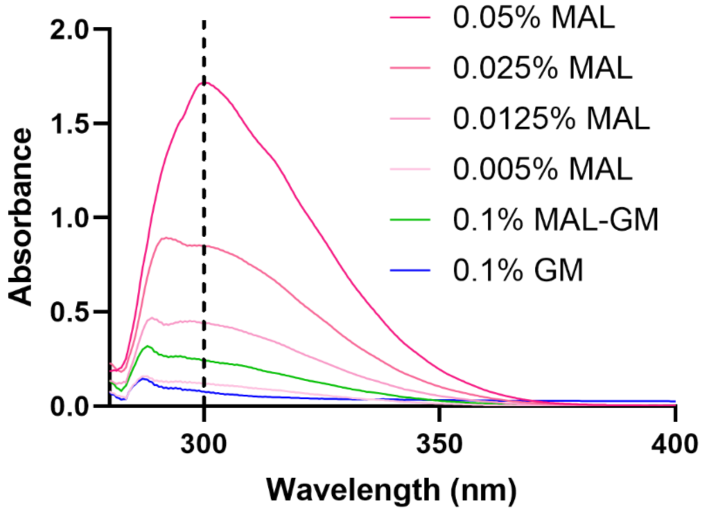


**Figure S3. UV-Vis spectrum of MAL-GM.** Maximum absorbance values of the maleimide group in both MAL-GM and 4-maleimidobutyric acid (MAL) occurred at a wavelength of 300 nm.

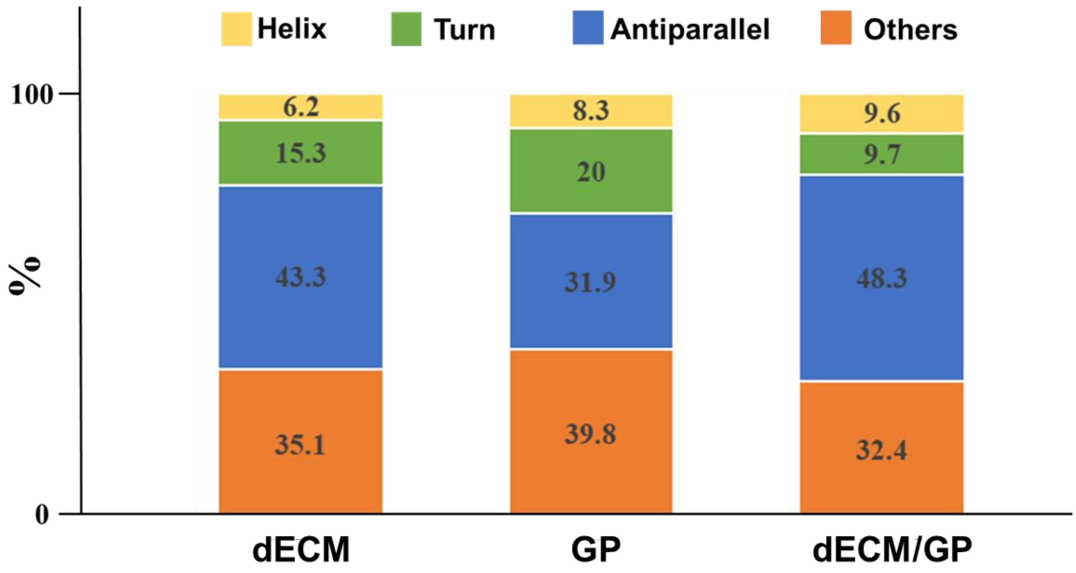


**Figure S4. Secondary structure prediction of dECM, GP and dECM/GP hydrogels.**

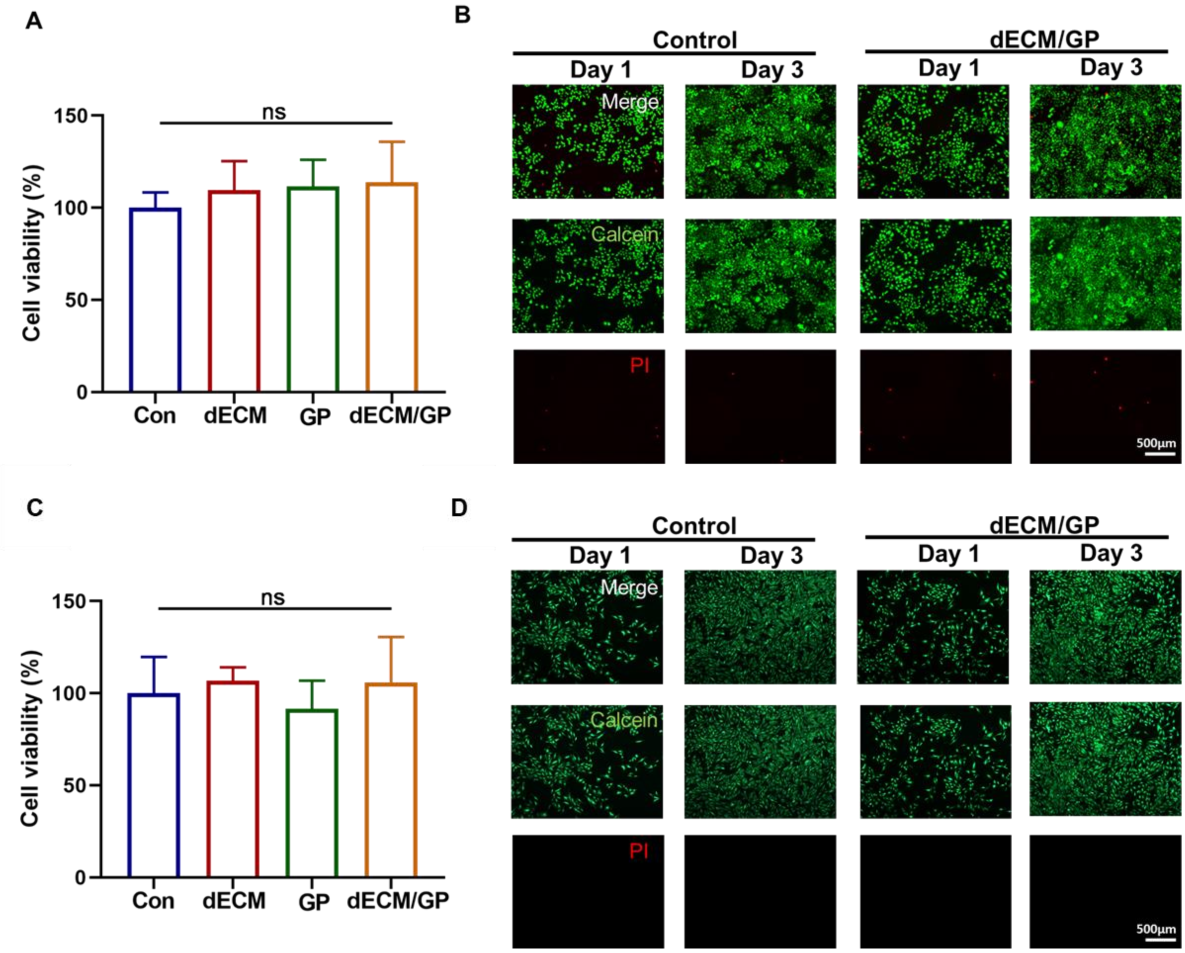


**Figure S5. Biocompatibility of dECM, GP and dECM/GP hydrogels co-cultured with L929 cells and H9C2 cells. (A)** L929 cell viability was evaluated by CCK-8 assay on Day 3 (n = 3). **(B)** Representative images of L929 cell Live/Dead staining. **(C)** H9C2 Cell viability was evaluated by CCK-8 assay on Day 3 (n = 4). **(D)** Representative images of H9C2 CELL Live/Dead staining. Viable cells were stained with calcein (green). Dead cells were stained with PI (red).

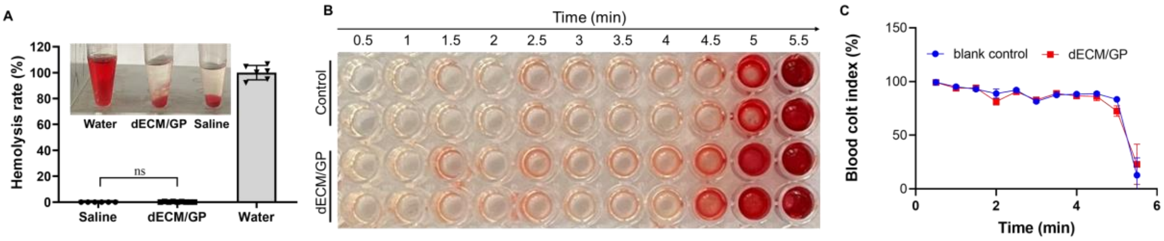


**Figure S6. In vitro blood compatibility of dECM/GP hydrogel. (A)** Hemolysis rate of (n=6 for saline and deionized water group and n = 9 for dECM/GP group.) **(B)** Thrombosis generated after being treated with blank control or dECM/GP. **(C)** Blood clot index of dECM/GP hydrogel (n = 6 for each time point).

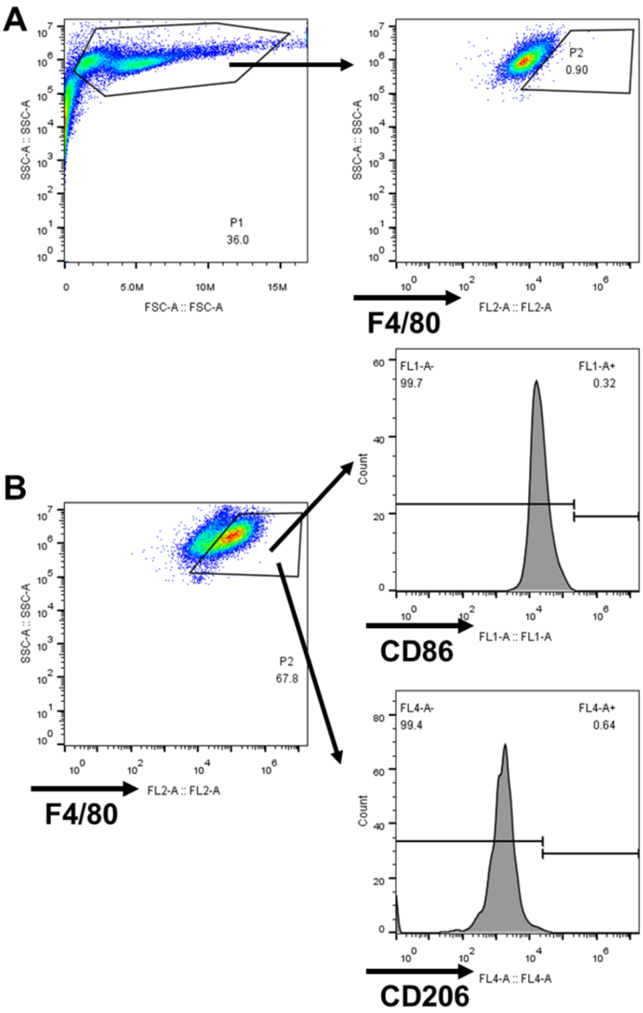


**Figure S7. Gating strategies for flow cytometry data. (A)** Gating strategy to select the F4/80 positive macrophages. **(B)** Gating strategy to determine the threshold value for CD86 and CD206 positive.

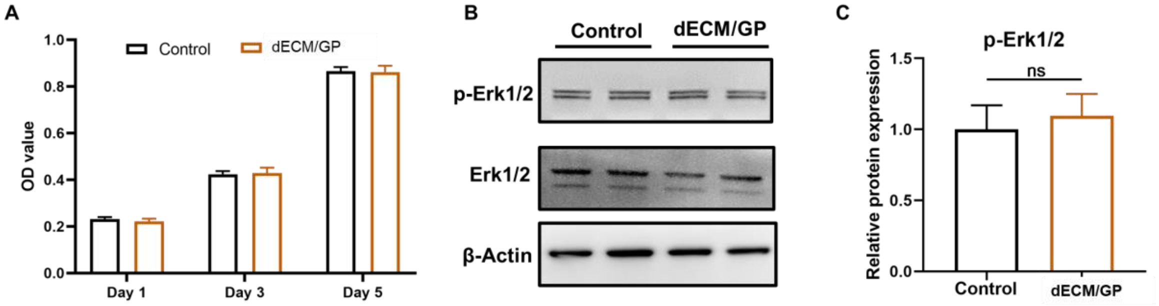


**Figure S8. Proliferation of HUVEC stimulated by dECM/GP hydrogel. (A)** Cell viability determined by CCK-8 assay (n = 3 for each time point). **(B, C)** Western blot analysis of p-Erk1/2 expression in HUVECs (n = 3).

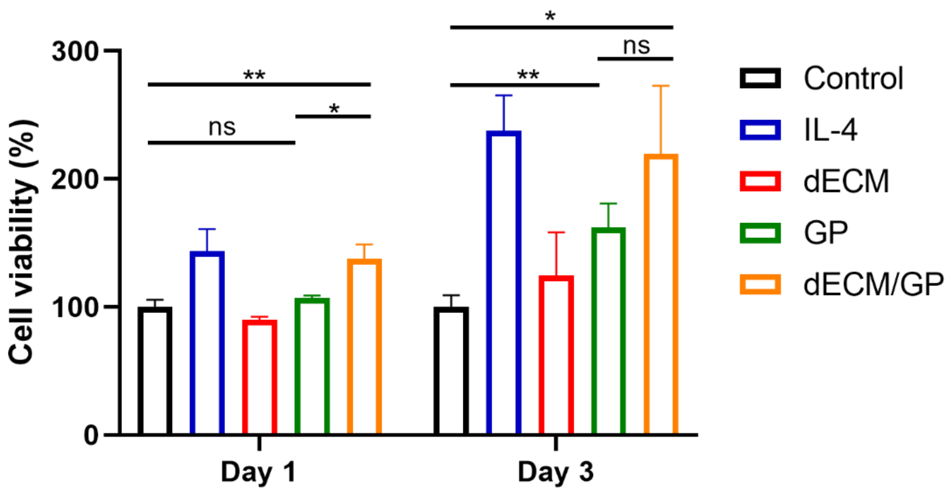


**Figure S9. Proliferation of MUVEC stimulated by coculture system with IL-4, dECM, GP and dECM/GP hydrogel treatment.** Cell viability determined by CCK-8 assay (n = 3 for each time point).

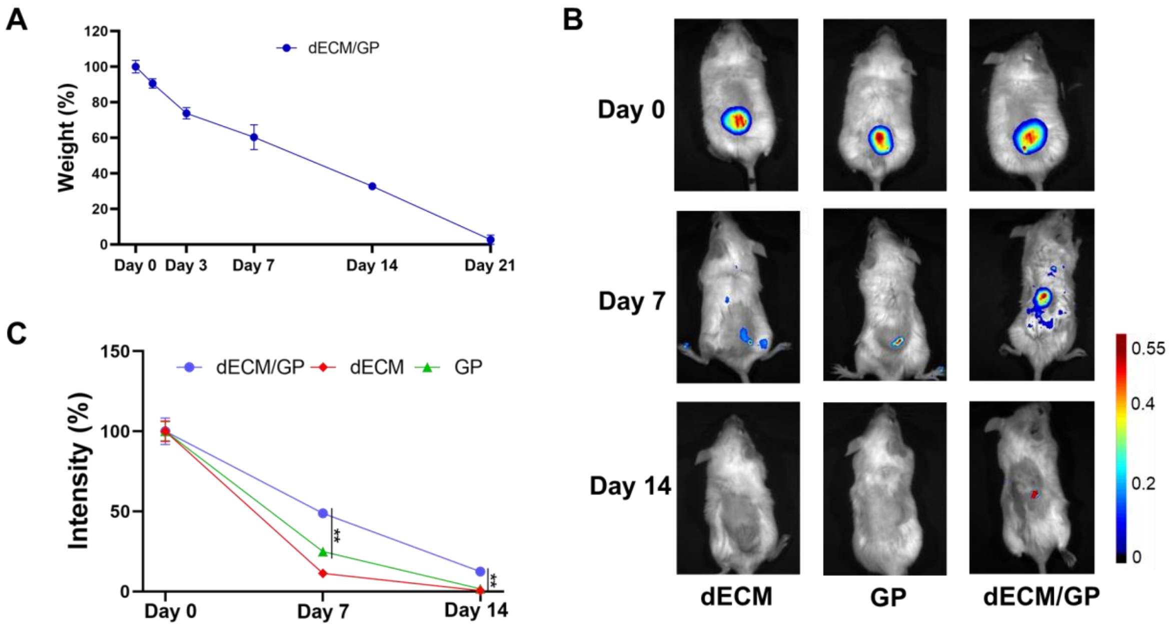


**Figure S10. In vitro and in vivo degradation behavior of dECM/GP hydrogels. (A)** In vitro degradation of dECM/GP hydrogel (n = 3). **(B)** Representative in vivo fluorescent images of mice after injection into the back of mice. **(C)** The fluorescence intensity at different time points after injection (n = 3).

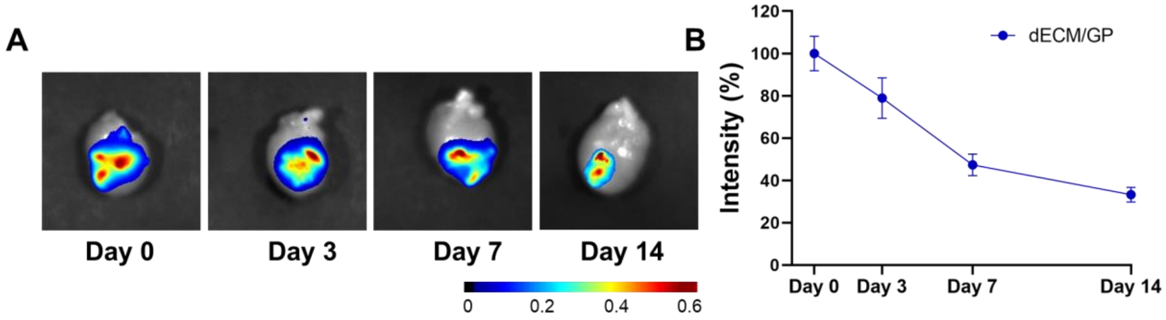


**Figure S11. Degradation of dECM/GP hydrogel in rat heart. (A)** Representative isolated fluorescent images of the harvested rat heart after injection of dECM/GP hydrogel. **(B)** The fluorescence intensity of rat heart after injection (n = 4 or 5).

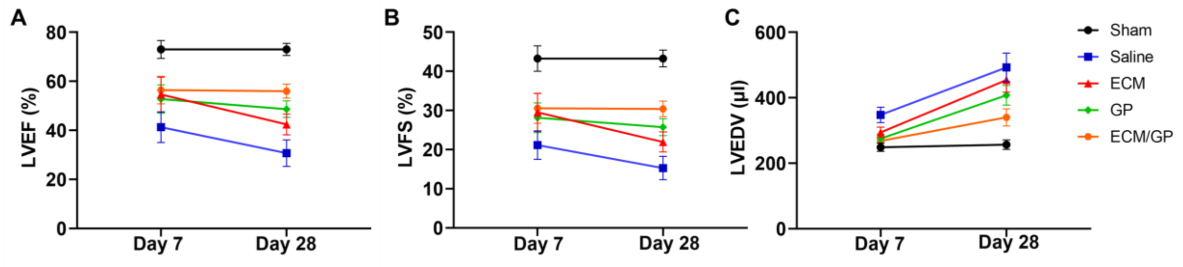


**Figure S12. Comparison of LVEF (A), LVFS (B) and LVEDV (C) on Day 7 and Day 28 (n = 7).**

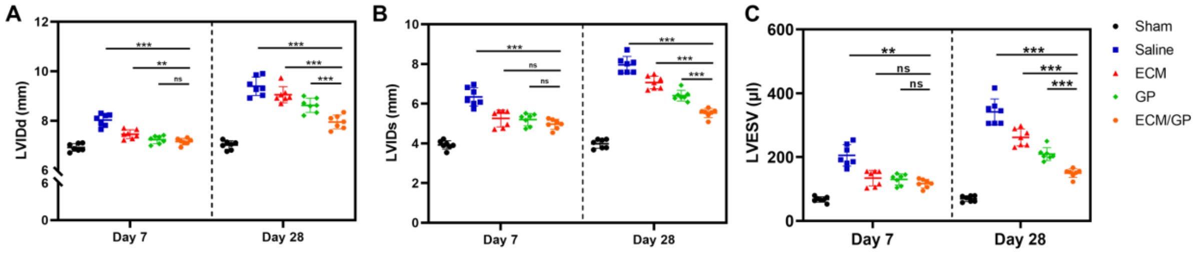


**Figure S13. Quantification of LVIDd (A), LVIDs (B) and LVESV (C) by echocardiography on Day 7 and Day 28 (n = 7).**

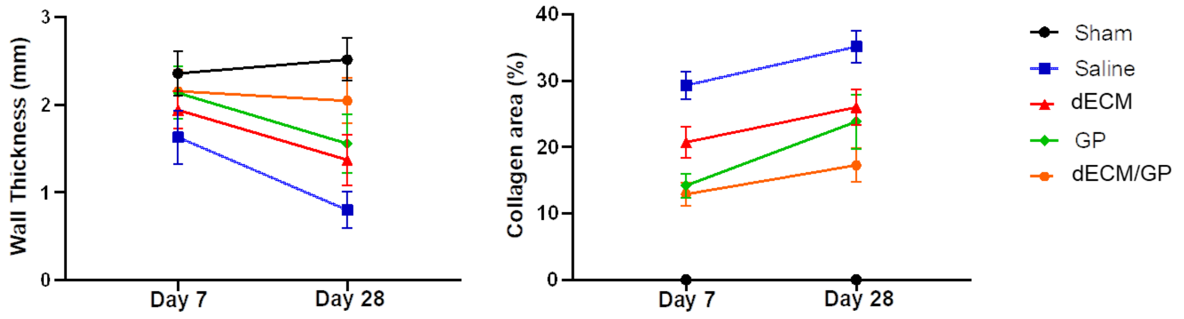


**Figure S14. Comparison of wall thickness and collagen area on Day 7 and Day 28 (n = 7).**

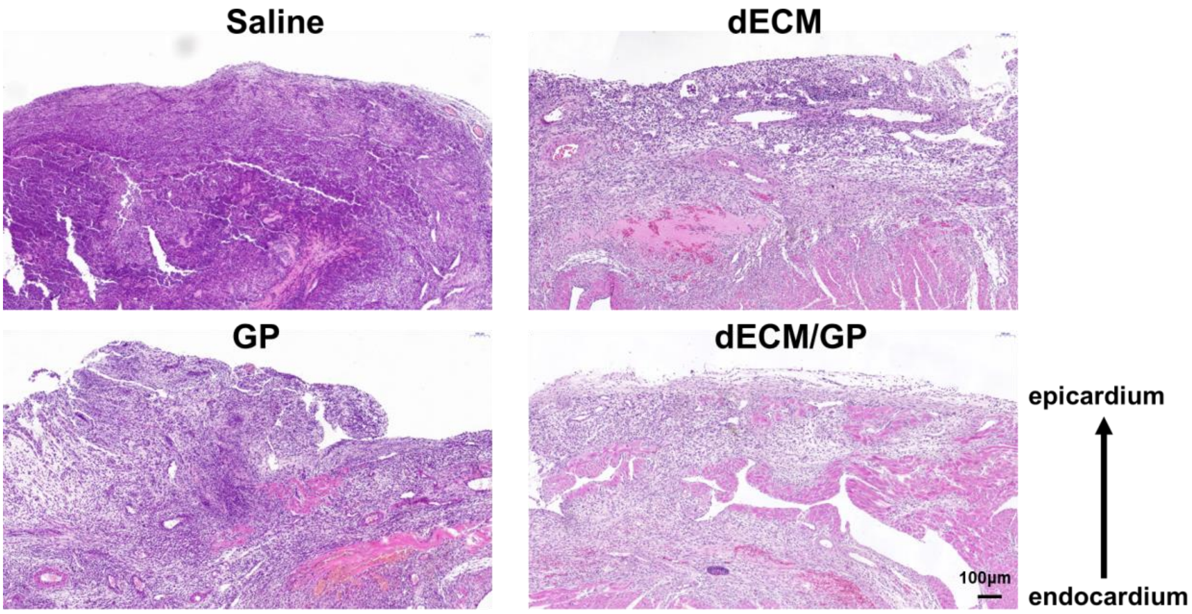


**Figure S15. Representative images of H&E staining of treated hearts on Day 7.**

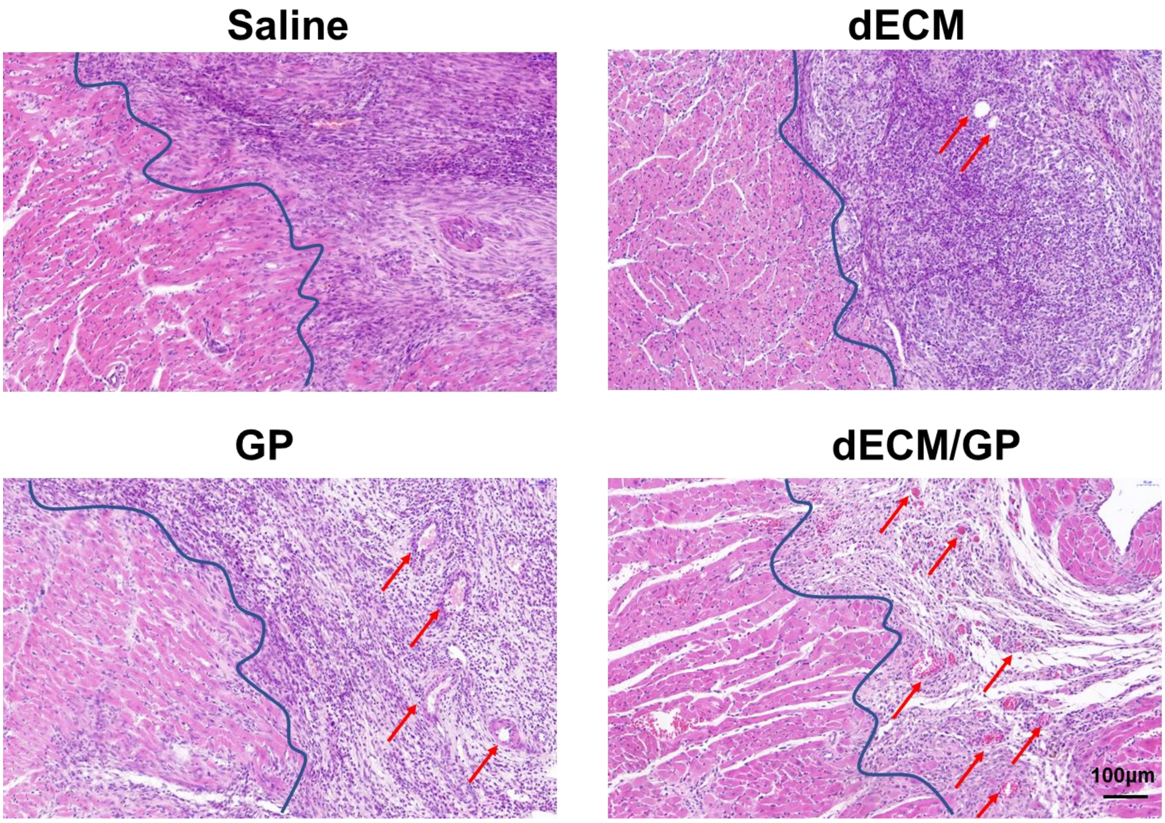


**Figure S16. Representative images of H&E staining of infarct and border area on Day 28.** Red arrows indicate small vessels.

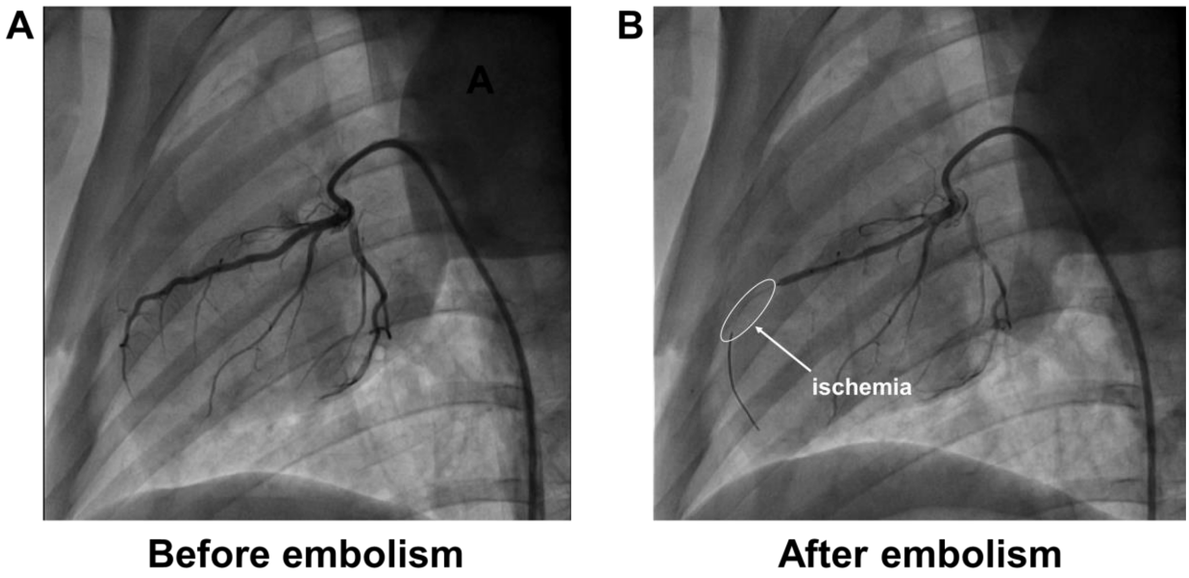


**Figure S17. Intracoronary embolization of mid left anterior descending coronary artery in a swine MI model.**

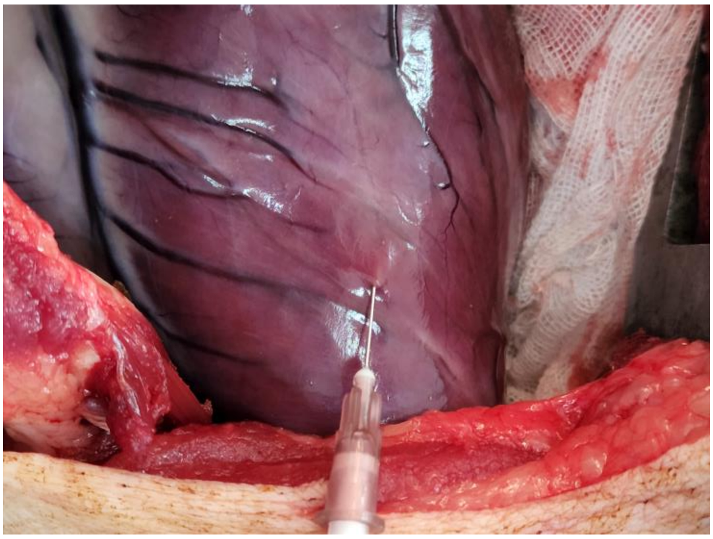

**Figure S18. Open-chest surgery and intramyocardial injection.**

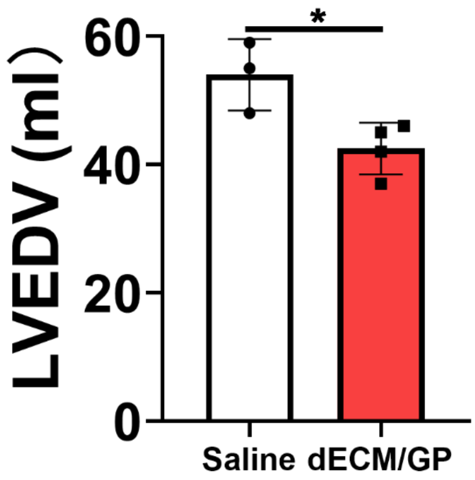


**Figure S19. Comparison of LVEDV in porcine MI model on Day 28.**

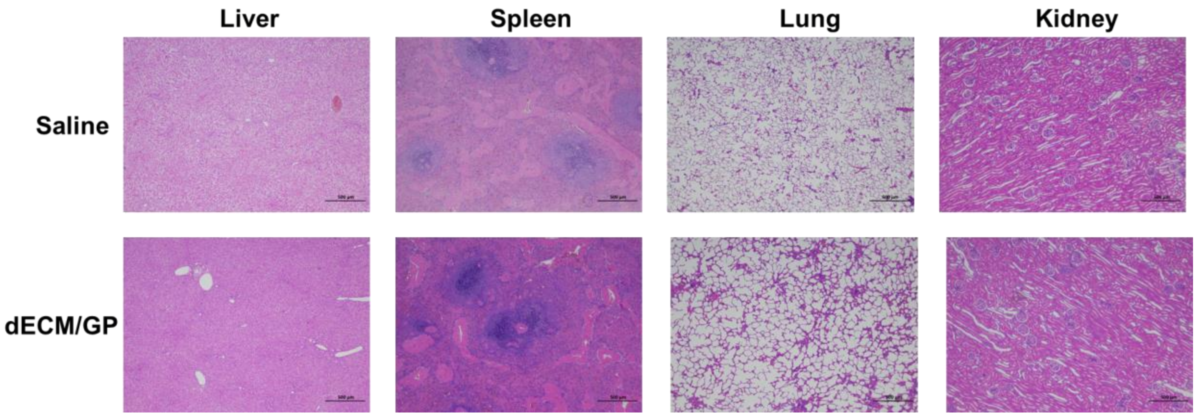


**Figure S20. H&E staining of liver, spleen, lung and kidney.**

**Table S1. Primer sequences**

| | |
|---|---|
| M-Arg1 Forward | 5'-ACATTggCTTgCgAgACgTA-3' |
| M-Arg1 Reverse | 5'-ATCACCTTgCCAATCCCCAg-3' |
| M-IL10 Forward | 5'-CCAAggTgTCTACAAggCCA-3' |
| M-ILl0 Reverse | 5'-gCTCTgTCTAggTCCTggAgT-3' |
| M-actin Forward | 5'-TCTGTGTGGATTGGTGGCTCTA-3' |
| M-actin Reverse | 5'-CTGCTTGCTGATCCACATCTG-3' |
| M-Vegfa Forward | 5'-TgggAgAACCCAAATgCTCC-3' |
| M-Vegfa Reverse | 5'-CACTAggCAACAgCACCTCA-3' |
| M-TGFβ1 Forward | 5'-ACTGGAGTTGTACGGCAGTG-3' |
| M-TGFβ1 Reverse | 5'-GGGGCTGATCCCGTTGATTT-3' |

**Table S2. Blood tests and liver and kidney function.**

| | Saline | ECM/GP | Normal range |
|---|---|---|---|
| **White blood cell ($10^3$/uL)** | 14.47±3.90 | 15.88±5.65 | 11.3-22.8 |
| **Red blood cell ($10^6$/uL)** | 6.67±0.81 | 6.95±1.53 | 5.9-8.8 |
| **Hemoglobin (g/L)** | 129.00±39.61 | 120.00±13.54 | 101-151 |
| **Platelet ($10^3$/uL)** | 185.67±29.48 | 209.25±78.36 | 138-467 |
| **Creatinine (μmol/L)** | 250.33±76.66 | 187.00±92.18 | 44-186 |
| **Albumin (g/L)** | 21.67±10.07 | 25.00±6.32 | 18-33 |
| **Alanine transaminase (U/L)** | 55.33±13.58 | 36.25±19.14 | 9-43 |
| **Aspartate aminotransferase (U/L)** | 26.67±17.16 | 45.50±20.63 | 16-65 |

**Data are shown as mean±SD.**

**Reference**

[1] A. K. Gaharwar, R. K. Avery, A. Assmann, A. Paul, G. H. McKinley, A. Khademhosseini, B. D. Olsen, *ACS Nano* **2014**, 8, 9833.

[2] Y. Hong, H. Song, Y. Gong, Z. Mao, C. Gao, J. Shen, *Acta Biomater* **2007**, 3, 23.
